# Individual objective versus subjective fixation disparity as a function of forced vergence

**DOI:** 10.1371/journal.pone.0199958

**Published:** 2018-07-06

**Authors:** Wolfgang Jaschinski

**Affiliations:** Leibniz Research Centre of Working Environment and Human Factors, Dortmund, Germany; University of Muenster, GERMANY

## Abstract

Inaccuracy in the vergence eye position (“fixation disparity”) can occur despite a fusion stimulus. When measured with eye trackers, this inaccuracy is referred to as “objective fixation disparity”. It is a matter of debate whether objective fixation disparity can be estimated with a technically simple psycho-physical procedure, i.e. the perceived offset of aligned dichoptic nonius targets, referred to as “subjective fixation disparity”. To investigate the relation between these two measures, simultaneous tests were made in far vision when placing prisms in front of the eyes (for a few seconds) in order to induce forced vergence, i.e. to vary the absolute disparity (from 1 deg divergent to 3.4 deg convergent). Frequent repeated measurements in 12 observers allowed for individual analyses. Generally, fixation disparity values and the effects of prisms were much smaller in the subjective than in the objective measures. Some observers differed systematically in the characteristics of the two types of prism-induced curves. Individual regressions showed that the subjective vs. objective slope was 8% on the average (with largest individual values of 18%). This suggests that sensory fusion shifts the visual direction of the (peripheral) binocular targets by the full amount of objective fixation disparity (since single vision was achieved); however, for the (central) monocular nonius lines this shift was more or less incomplete so that the dichoptic nonius targets indicated an individual percentage of objective fixation disparity. The subjective-to-objective ratio seems to be an individual characteristic of fixation disparity in terms of the amount and in terms of the effect of prism-induced forced vergence. Therefore, on the group level the subjective measures do not allow for a precise prediction of the objective measures.

## Introduction

Viewing with two eyes has the advantage that the images in the two eyes are integrated for better visual performance and for stereoscopic vision [1–3]. This is achieved by several steps of physiological mechanisms. Basically, in a first step neural activity adjusts the external eye muscles in order to reach an angular position of the two eyeballs where the foveola in each eye is directed as precisely as possible towards the visual target (oculomotor fusion). In further steps, neural processing in the visual cortex provides the best possible integration of the two images in the brain (sensory fusion). Also in normal binocular vision, smaller errors in oculomotor and/or sensory fusion may occur without problems in these observers. However, larger errors can reflect fusional difficulties in individual subjects which may lead to eye strain, visual discomfort and also reduced stereo vision. For the diagnosis of binocular disorders, several procedures are available, including tests of fixation disparity, heterophoria, and stereo vision.

The present study focuses on a phenomenon called fixation disparity. It refers to binocular errors (disparity) during fixation of a visual target with both eyes as in natural vision; accommodation is involved as well. Fixation disparity has both oculomotor and sensory components. The quantitative relation of these two components is still an open question and was therefore investigated in the present study. Our recent papers [4, 5] have provided comprehensive literature reviews of fixation disparity in terms of their amount, nature and interpretation. The present introduction provides a concise summary of the current state of research. This will lead to the aims of the present study and the hypotheses.

### Definition of objective versus subjective fixation disparity

**Fig 1:**
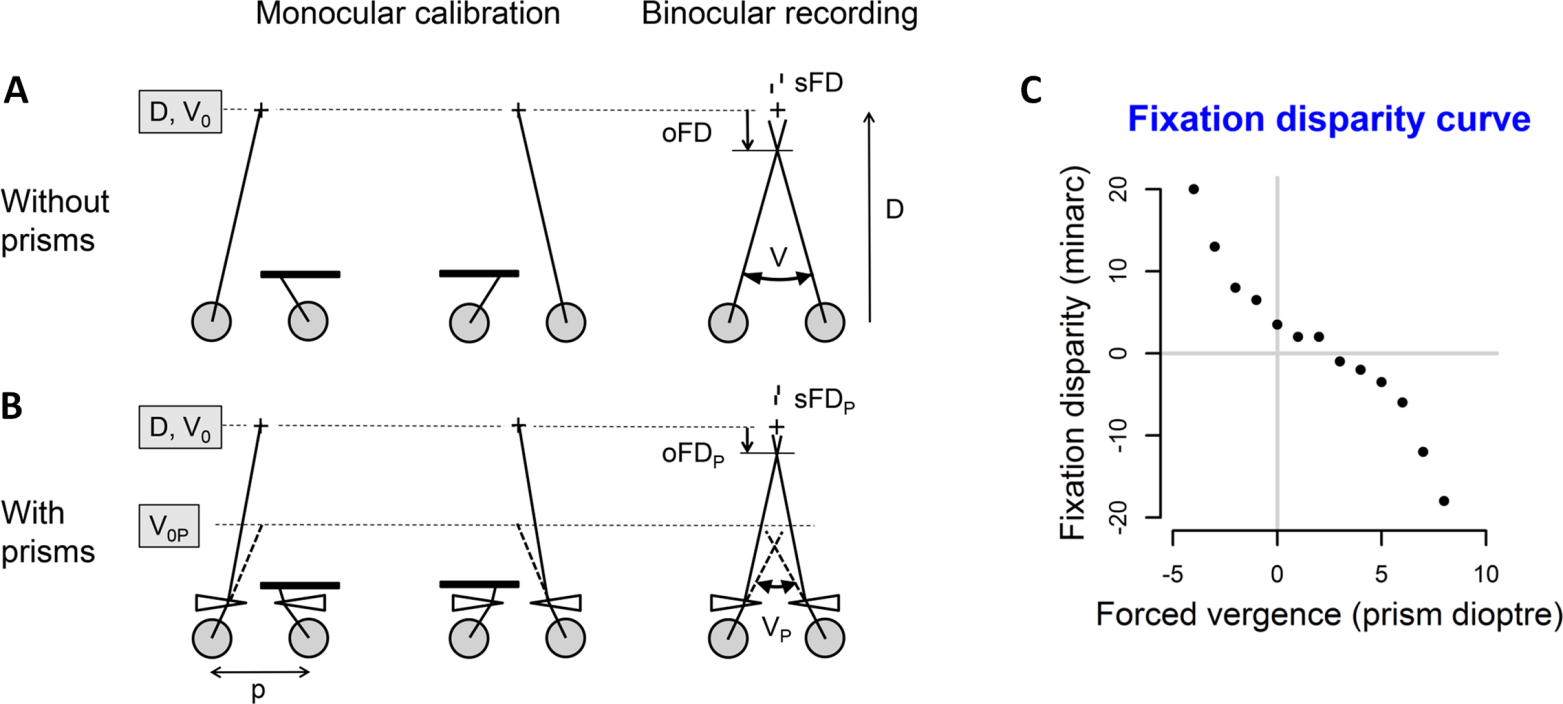
Conditions of measuring fixation disparity. (A) Without prisms, the objective fixation disparity (oFD) is the difference between the observed vergence angle V during binocular recording and the stimulus vergence angle V_0_, which is geometrically given by the viewing distance D and the interpupillary distance p, i.e., V_0_ = 2 arctan (p/2D). In this example of an over-convergent (eso) oFD, V is larger than V_0_. The monocular components of V_0_ are measured during the eye tracker calibration that is made separately for the left and right eye: the eye position during monocular fixation represents the zero position for the subsequent binocular recording period. The covered eye assumes the heterophoria resting state. For an optical correction of an eso fixation disparity (as in this example), base-out prisms are applied. These prisms turn the visual axes optically outward (drawn lines), which requires the eye muscles to converge more (broken lines) to maintain fusion. (B) When prisms are applied, V_0P_ = Prismpower + V_0_ is the stimulus vergence angle and V_p_ is the vergence angle. The subjective fixation disparity is illustrated by the angular amount of horizontal offset d_Non_ between two dichoptically presented nonius lines (sFD = arctan (d_Non_/D), d_Non_ > 0); this offset has to be adjusted on the test monitor so that the observer perceives the two nonius lines in alignment. Subjective fixation disparity is typically smaller than the objective fixation disparity, as indicated in the graph. The graphs show the case of visual axes that intersect in front of the fixation point when the fixation cross is projected on the nasal part of the retina within Panum’s area; this over-convergent state is referred to as eso fixation disparity with a positive sign. In the opposite under-convergent state, the visual axes intersect behind the fixation point, the fixation cross is projected on the temporal part of the retina within Panum’s area and the nonius lines have a reversed position (d_Non_ < 0, exo fixation disparity with a negative sign); in the latter case, base-in prisms are applied. (C) Schematic diagram of a typical “fixation disparity curve”, i. e., fixation disparity is plotted as a function of forced vergence when prisms are placed in front of the eyes. Conventionally, positive signs are used for base-out prisms and negative signs for base-in prisms.

Objective fixation disparity (oFD) measures the vergence error by comparing the visual axes when the target is viewed monocularly and then binocularly (see Fig 1). The visual axes may optimally intersect at the fixation point; in other conditions, they may intersect behind or in front of, which is referred to as exo or eso fixation disparity, respectively. Essential for calculating oFD is the definition of oFD = 0 which is related to the monocular calibration procedure of the eye tracker. During calibration, the observer fixates a monocular calibration target which is assumed to be projected onto an assumed centre of the fovea. If this position in each eye holds also for binocular vision, the objective fixation disparity is zero.

The subjective fixation disparity (sFD) refers to the test result of a perceptional phenomenon when viewing two nonius targets that are presented separately to the left and right eye (in the vicinity of a binocular fusion target). If these two “dichoptic” objects are physically in line, they may nevertheless be perceived with a horizontal offset. In earlier studies, this psychophysical test result was understood to indicate the position of the visual axes, as it is shown, e.g., in the classical geometric illustration of Ogle [6]. This common understanding might have originated from clinical measures of vergence when no fusion target is involved: the resulting heterophoria can veridically be measured based on the perceived offset of non-fusible targets in the two eyes, e.g., with a point and streak of line in the Maddox procedure [7]. If–in non-fusion conditions–dichoptic nonius targets fall on corresponding points on the retinae, the targets appear in line and their physical horizontal distance indicates the vergence angle.

From the definition in Fig 1, it appears to be theoretically clear what a zero fixation disparity means. Physiologically, however, different views have been proposed, depending on the neurophysiological models that are assumed. Feedback control models understand the fixation disparity as a purposeful error that is required for maintaining a stationary vergence state [1, 8]; accordingly, a state of zero fixation disparity should not exist. In the neural network model of Patel et al. [9–11], fixation disparity results from an imbalance between the convergent and divergent pathway with different gains; accordingly, zero fixation disparity can occur if both pathways have the same gain. Experimental studies provided evidence that zero fixation disparity can occur both for the subjective measure and for the objective measure [4, 12–14]. Further aspects of zero fixation disparity are described in the Discussion.

### Fixation disparity depending on vergence stimulus

In natural vision, the vergence state changes with the viewing distance: on average, the subjective fixation disparity changes from an eso state in far vision towards an exo state in near vision, while such a general trend seems not to exist for objective fixation disparity [4, 15, 16]; for both measures, individuals differ in the effect of viewing distance. A technically easy way to vary the vergence state is to apply prisms in a trial frame in order to force the eyes to adopt an intended vergence state: base-out prisms force the eyes to converge, while base-in prisms force the eyes to diverge, both relative to the baseline vergence state corresponding to the current viewing distance [6, 17]. The accommodative stimulus, however, is not varied when prisms are applied; the resulting vergence-accommodation mismatch will stimulate vergence adaption. A typically resulting “fixation disparity curve” is depicted in Fig 1C: the function is more or less flat and rather linear with small prism load (in the centre of the curve), but the slope increases in the negative (exo) or positive (eso) direction when the prism load approaches the fusional limits, i.e., the point where double vision occurs. The typical curve is monotonic with a generally negative slope. Steep subjective fixation disparity curves tend to indicate a higher level of asthenopic complaints, both as a function of prism-load [17] and as a function of viewing distance [18].

While subjective fixation disparity curves have widely been investigated and are clinically relevant [19, 20], research on objective fixation disparity curves is very limited since elaborate techniques for eye movement recording are required for detecting the small amounts of objective fixation disparity. The following paragraph reviews the two types of fixation disparity as a function of forced-vergence.

### Relation between the two types of fixation disparity curves

The early study of Hebbard [21] in 1962 had reported agreement between subjective and objective fixation disparity (in the one observer tested), while research since about 1985 provided increasing evidence that dichoptic nonius lines do not measure the correct amount of the vergence error when fusion stimuli are involved [12, 13, 22–32]. It was concluded that the mechanism of binocular fusion affects the perceived visual direction of monocular nonius lines; but the stimulus conditions play a role.

Although seven research groups reported differences between sFD and oFD, surprisingly only Remole et al. [22–25] investigated the quantitative relation between these two measures on the individual level. In his first studies [22–24], Remole did not use an eye tracker, but he estimated the oculomotor position of the visual axes from a perceptional phenomenon, i.e. the horizontal width of perceived enhanced contrast of a Mach band near a vertical black-white border. The width of this “perceived border enhancement” increases with retinal eccentricity and is not affected by changes in retinal correspondence as it is the case for dichoptic nonius lines. Remole called this measure “fixation misalignment”, however it will be referred to in the present paper as “estimated objective fixation disparity” in relation to eye tracker studies where objective fixation disparity is measured. Remole described the relation between this estimated objective fixation disparity and the subjective fixation disparity (sFD) by the “projection change ratio”, which he defined as

$${PCR}_{i}=\frac{\left(estimated\;o{FD}_{i}-{sFD}_{i}\right)}{{estimated\;oFD}_{i}}=1-\frac{{sFD}_{i}}{{estimated\;oFD}_{i}}$$

The index i denotes the individual subject. The amount of PCR was in the range of 0.85–0.95 when forced vergence was up to about 30 prism diopter (cm/m).

The present study will use a slightly different formula that describes the subjective fixation disparity (sFD_i_) of an observer i as a percentage R_i_ of objective fixation disparity (oFD_i_), i.e.

$${sFD}_{i}=R_{i}*{oFD}_{i},\;thus\;R_{i}=1-{PCR}_{i}$$

This formula was chosen since it is known that sFD may be zero despite a considerable amount of oFD, and in other conditions sFD may be as large as oFD; thus, the expected R_i_−values range between 0 and 1. This formula reflects earlier findings [20, 25] that sFD is the result of two physiological mechanisms: (1) the motor vergence error (measured as oFD) and (2) the sensory processing of the visual directions of the nonius lines (which cannot be measured directly, but may be represented by the factor R_i_).

**Fig 2:**
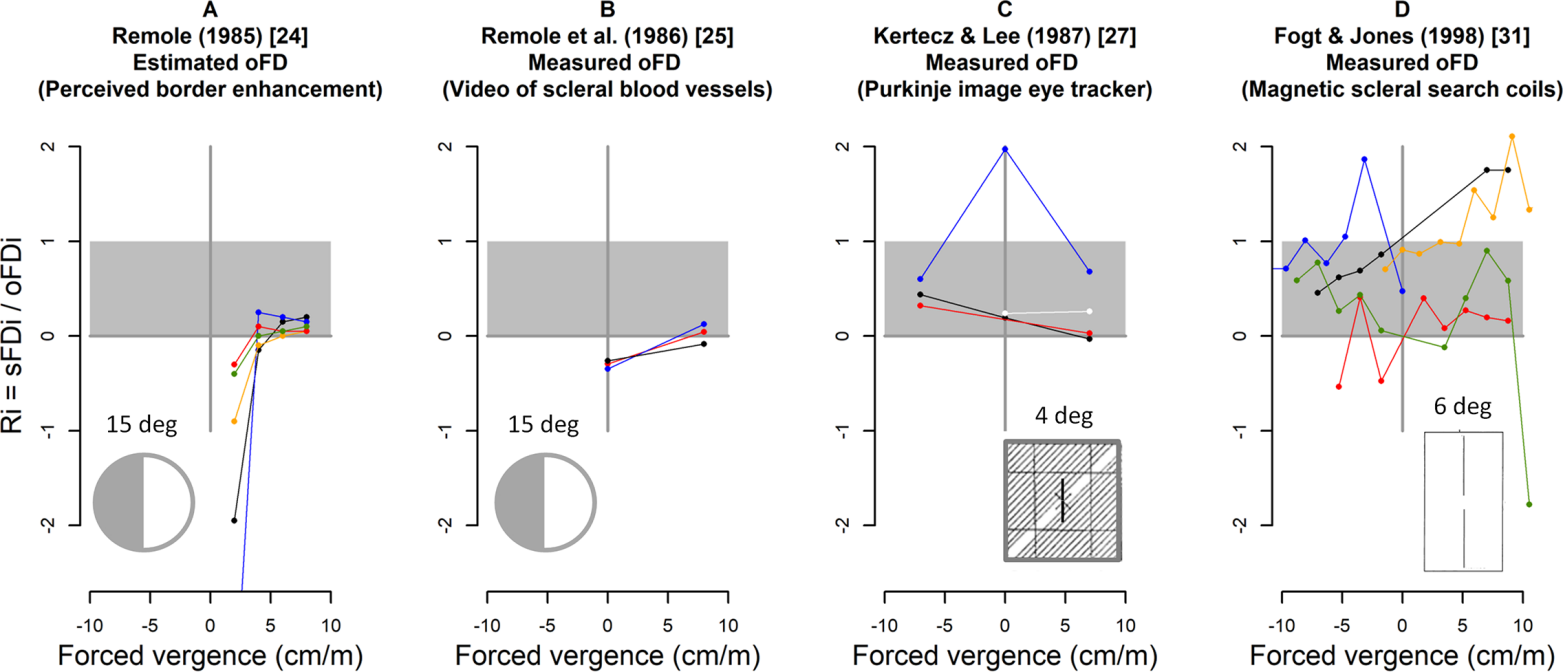
Individual ratios R_i_ = sFD_i_ / oFD_i_ as a function of forced vergence (prism dioptre = cm/m), re-analysed from published data. Different colors represent data of different individuals; the studies had different participants. Remole’s [24] “projection change ratio” = 1 –(sFD_i_/oFD_i_) = 1 –R_i_ was converted to R_i_ = sFD_i_/oFD_i_, based on the “estimated oFD” (perceived border enhancement) in Fig 2A. Remole had measured the perceived border enhancement up to forced vergence of about 30 prism dioptre and found constant PCR–values; these are omitted in this replotted graph. Fig 2B shows the complete data that Remole et al. [25] measured objectively using video recording of retinal blood vessels. Fig 2C and Fig 2D refer to the published sFD_i_ and oFD_i_ data of Fogt and Jones [12] who used magnetic scleral search coils and of Kertez and Lee [27] who used a Purkinje image eye tracker. The gray area indicated the range of expected R_i_-values; see Introduction. A condition of R_i_. = 1 would mean sFD = oFD; this was generally not observed. Note that the R_i_ = sFD_i_/oFD_i_ values inflate toward infinity if oFD_i_ approaches zero. The resulting outliers with abs (R_i_) larger than 2 were omitted; these were 5% of the data in Fig 2A, 17% in Fig 2C, 18% in Fig 2D; Fig 2B had no outliers.

Fig 2 illustrates the individual ratios R_i_ as a function of forced vergence from those previous studies where these ratios could be calculated from the published data. These Ri−values where not calculated and reported by these authors, but note that Remole plotted the PCRi−values. Fig 2A shows the R_i_−values based on Remole’s border enhancement technique [24]: when forced vergence was 5 cm/m and larger, the R_i_−values were small and positive in the range of 0–0.2 (i.e., sFD is about 10% of oFD), while towards zero forced-vergence surprisingly large negative R_i_−values occurred (see Discussion). Fig 2B shows R_i_−values of Remole et al. [25] based on objective video recordings of scleral blood vessels: with forced vergence, R_i_−values closely scatter around zero, while without forced-vergence they are moderately negative at about -0.3. Fig 2C shows the data of Fogt and Jones [12] where most R_i_−values scatter in a wide and mostly positive range up to 2. This is roughly confirmed by the smaller dataset of Kertesz and Lee [27] shown in Fig 2D. Obviously, most data deviate from R_i_ = 1 and–with some exceptions–the Ri−values are smaller than 1, thus sFD is smaller than oFD. But–surprisingly–also negative R_i_ –values occur.

### The correlation between the two types of fixation disparity

Regression analyses between subjective and objective fixation disparity in groups of subjects showed a moderate correlation of about r = 0.5 between the two types of fixation disparity without forced vergence [13]: the subjective fixation disparity was much smaller than the objective fixation disparity in most cases; even the sign can be different at small amounts of fixation disparity. However, such a group-level correlation will be low, if individuals differ in the relation between subjective and objective fixation disparity. Therefore, the present study investigates this regression for each participant i across many single recordings j:

$${sFD}_{ij}=k_{ij}+C_{i}*{oFD}_{ij},\;for\;each\;individual\;i$$

Note, that–as in every regression–a constant term k_i_ is included, although–theoretically–one would expect sFD = 0 at oFD = 0. Such an individual regression requires frequent repeated single measurements j of sFD and oFD per individual i and a certain variation of fixation disparity so that a physiologically existing relation can statistically be found given the inevitable measurement error. In the present study, the experimental variation of fixation disparity within individuals was reached by modifying the amount of the vergence stimulus, i.e., the absolute disparity of the stimuli.

### Aim of the study

It was intended to compare individual objective and subjective fixation disparity curves for a quantitative comparison. Therefore, video eye tracker recordings were made in 12 observers. Forced vergence was applied with prisms in the range from 2 prism dioptre base-in to 6 prism dioptre base-out. The analyses comprised several steps.

1. Individual FD-curves were described by their y-intercept (fixation disparity at zero forced vergence) and by their slope (effect of forced vergence on fixation disparity).
2. The intra-individual regression coefficients C_i_ between the two types of fixation disparity were calculated from the individual sFD–oFD regression including the variability due to the varied amount of forced vergence.
3. On the group level, subjective-versus-objective regressions for the y-intercept and the slopes were made.
4. The regression coefficients C_i_ were compared with two individual ratios, i.e. with the y-intercept ratio R_0i_ = sFD_0 i_ /oFD_0 i_ and with the slope ratio R_slope i_ = sFD_slope i_ /oFD_slope i_.
5. The ratios R _i_ = sFD _i_ /oFD _i_ were plotted as a functions of the amount of forced vergence, for comparison with analyses made in Fig 2 based on earlier published data.

The findings suggest that the subjective fixation disparity reflects a small, individual proportion of objective fixation disparity. Potential physiological mechanisms are discussed. The condition of zero fixation disparity requires particular consideration.

## Methods

The methods resemble those in our previous study [5]. Stimuli appeared at 5 m viewing distance on a 3D-television monitor (LG 32 LW 4500, with circular polarization) in order to use dichoptic nonius targets for measuring subjective fixation disparity. Eye movements were measured with the EyeLink II system (SR_Research), however in a modified way for precise recording of the small amount of objective fixation disparity below 2 deg [13]; many details have been reported by Schroth et al. [5] and Jaschinski [4].

### Stimuli and apparatus

**Fig 3:**
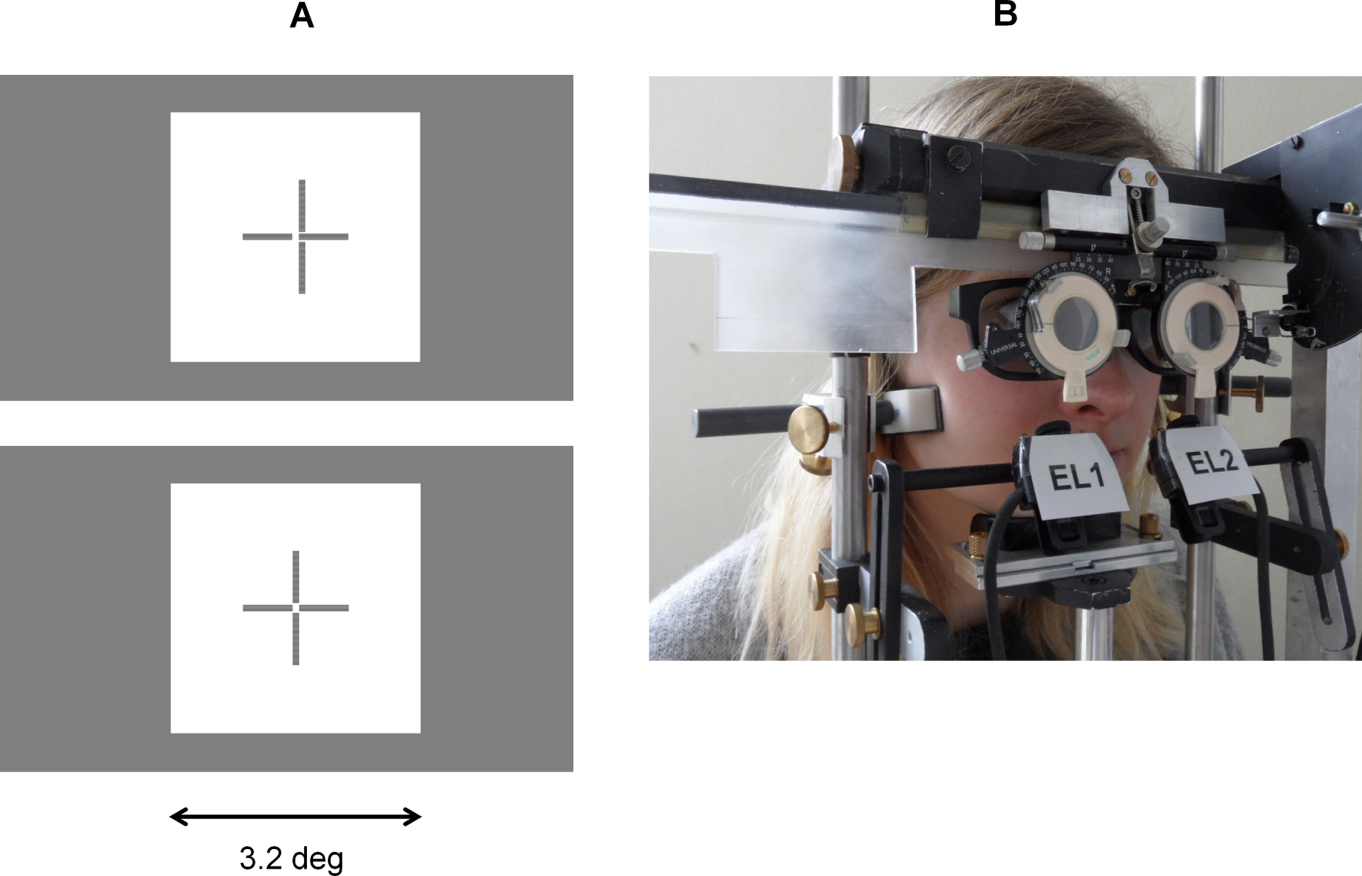
Illustration of stimulus and experimental setup. (A) The stimulus comprising a peripheral frame with horizontal fusion contours at ± 1.6 deg and dichoptic nonius targets with central gaps; the vertical lines were visible for the right eye, the horizontal lines for the left eye. (B) Observer in the adjustable headrest with rests for chin and forehead; the cheekbones were fixed to prevent horizontal head movements; a flexible band around the head held the observer in the headrest. Polarizors provided dichoptic viewing of the nonius targets. The EyeLink II cameras had a free view of the eyes below the filters and prisms. An occluder was shifted in front of each eye for the monocular calibrations.

Fig 3A illustrates the structure and the dimensions of the stimulus that comprised a central cross with dichoptic nonius lines and peripheral quadratic frame with horizontal fusion contours at ± 1.6 deg. The square had a luminance of 90 cd/m^2^ and the screen background 20 cd/m^2^, as measured through the polarizors. Subjective fixation disparity was measured with central dichoptic nonius targets, i.e., a pair of horizontal lines was visible for the right eye and a pair of vertical lines was visible for the left eye. Both pairs of lines had a central gap. The observer adjusted the vertical lines horizontally with the left and right button of a computer mouse, so that the vertical lines crossed the horizontal lines at the gap; see Fig 3A for misaligned and aligned conditions. This “Cross-test” was proposed by Haase [33] as part of the “Measuring and Correcting Methodology after H.-J. Haase (MCH)” [34] that suggests prisms for constant wear to reduce asthenopic complaints [35, 36]. These tests are primarily applied in far vision. The Cross-Test is the first in the series of MCH-tests and is supposed to identify the oculomotor component of fixation disparity [37].

Fig 3B shows the apparatus for the application of prisms and for keeping a fixed head position; the latter is required for precise eye movement recordings. A headrest was equipped with a movable frame for trial lenses where prism eye glasses could be inserted. This frame could be flipped up and down manually to allow vision without and with prisms, respectively. A chin and forehead rest, a band around the head and narrow temporal rests were applied to minimize artifacts on eye movement recordings due to possible lateral and oblique head movements; a bite bar was not used. The headrest could be adjusted in order to place the eye at a defined position for all subjects. This correct eye position was controlled by a video camera beside the head. The two EyeLink II cameras were fixed to the headrest. An opac occluder could be shifted in front of the left and right eye for the monocular calibration.

### Procedures of measurements

#### A single recording

A single recording for a one-minute data collection was made as follows. (1) For the calibration of the left and right eye, the observer fixated monocular fixation crosses and pressed a button when he/she was precisely fixating; these data were stored. (2) In the series of nonius adjustments, the observer shifted the vertical lines to perceived alignment relative to the centre of the horizontal lines by using the left and right button of the computer mouse. When subjective alignment was reached, the observer clicked the centre computer mouse button. The resulting nonius offset was recorded as a single data of the subjective fixation disparity and the corresponding eye position and pupil size were recorded. To avoid artefacts due to blinking in the moment of clicking, across the interval 100 to 400 ms before clicking the median and standard deviation of objective fixation disparity was calculated as a single data. The median of these standard deviations was 2.1 minarc. In 3% of all single data the standard deviation was larger than 10 minarc; these single data were discarded.

One nonius adjustment took about 3 seconds. This was made first without a prism. When the experimenter noticed the mouse click of the observer, she flipped a pair of prisms in front of the eyes and the observer immediately started the next nonius adjustment (with a randomly chosen starting offset). In this way, adjustments without prisms and with prisms were made alternatively. (3) After a 1 minute period of data collection, the monocular calibration was performed again as described above. In this way, prisms were placed in front of the eyes repeatedly for a few seconds only and removed again. This is the classical procedure for measuring fixation disparity curves in order to eliminate prism adaptation [20].

#### Measurement of a fixation disparity curve

A series of 5 single recordings was made, each with a different amount of prism load. The order of conditions was 0, -2, 2, 4, 6 prism dioptre, in order to start the series with natural vision and to have the higher vergence load at the end of the series (to avoid potential effects on subsequent recordings). Between the single recordings, a rest pause of 3 minutes was applied.

Three observers were not able to fuse the stimulus in the full intended range of prism load. Therefore, Participant 07 did not receive 6 prism dioptres and Participants 08 and 09 did not receive 4 and 6 prism dioptres. Also in the remaining subjects, some single large subjective fixation disparity data may question fusion. However, the individual curves in S1 Fig show that the robust linear regression basically relies on subjective fixation disparity data that are smaller than 15 minarc. This procedure confirms that the present results describe the state of fusion.

#### Design of the study

Two experimental sessions were made on different days. Each session comprised two subsequent measurements of a fixation disparity curve, with a rest pause of 20 min between the two series per session. A further session comprised a complete optometric investigation of the observers’ vision as reported above.

### Eye movement recordings

The video-based EyeLink II (SR Research Ltd, Osgoode ON, Canada) was used with the dark pupil detection mechanism that tracks the centre of the pupil. Recorded data were analyzed based on the raw data, sampled every 2 ms (500 Hz). The filters of the EyeLink software were switched off. The conventional EyeLink II procedures were modified in order to improve performance for measuring fixation disparity; the accuracy of the present recording and measurement approach are fully described in Jaschinski [4] and Schroth et al. [5]. In short, the recording system has a physical resolution of 0.6 minarc. In order to reduce errors introduced due to calibration and during recording, the present procedure used a short 1 minute recording period with a pre- and a post calibration, a rigid head stabilization, a series of repeated measurements that were averaged to reduce random error. In the end, the quality of the data can be estimated from the reported individual confidence intervals.

Instead of the original EyeLink II calibration mode, we used the raw data and applied the following monocular calibrations before and after the 1-minute recording period that were averaged. The use of polarizors is not sufficient for complete monocular vision during the calibration since the mechanical frame of the OLED display may be effective as peripheral fusion target. Therefore, the right eye was covered with an opal occluder for calibrating the left eye and, subsequently, the left eye was covered for calibrating the right eye. The opal occluder was chosen to make all stimuli invisible, but to lower the luminance by only 30% so that the pupil dilated only slightly due to the occlusion. For calibration, subjects were requested to carefully fixate one of three calibration targets (crosses of 14 min arc) that appeared sequentially in the screen centre (zero position) and left and right horizontal positions of 2.3 deg. Each of the three calibration targets was presented twice in random order to average across variability in fixation.

Video eye trackers detect the centre of the pupil and therefore assume that the pupil centre keeps its position if the eyes do not move. But, variation in pupil size can lead to horizontal and vertical shifts of the pupil centre [38]. A 1 mm dilatation of the pupil induces a 27 minarc exo shift in the measure of objective fixation disparity (average across observers). This pupil artefact is relevant whenever the pupil diameter varies in a systematic way in an experiment. In the present study, it was found retrospectively that the pupil was generally larger when prisms were placed in front of the eyes. The reason was the non-transparent circular frame of the prisms that restricted the visual field to a 20 mm aperture. Therefore, less ambient room lighting was visible in the prism condition and the pupil was larger by 0.10 ± 0.13 mm (mean ± SD, range—0.28 to 0.15) in the prism conditions (-2, 2, 4, and 6 prism dioptre) than in the 0 prism condition. To account for a potential artefact, a correction was made with a procedure suggested by Jaschinski [38]: based on individual regressions of oFD as a function of random variation in pupil size, it was calculated an corrective value of objective fixation disparity for each of the present participants. Thus, in the prism conditions, the oFD-values were shifted by individual corrections that ranged from 5.82 to -2.06 minarc (mean ± SD, 2.21 ± 2.27 minarc); thus, the correction was mostly in the eso direction. This 7.88 minarc range of these corrections was 11% of the 70.89 minarc range of the corrected values of objective fixation disparity. This suggests that this correction was marginal for the final results in the present conditions. Note that a pupil correction is not required for the subjective fixation disparity data since they are measured with nonius lines.

### Data analyses and statistics

Given the typically marked individual differences in binocular vision, data were first analysed separately for each observer. Then analyses across participants were performed. The analyses and the graphs were made with the open-source software R [39].

On the individual level, for each of the four measurements of a fixation disparity curve (two in each of the two sessions) all single data of subjective and objective fixation disparity were used for regressions analyses. In a first step, 3^rd^ order polynomials were fitted to all data of each of the four curves since this polynomial represents the generally expected function (Fig 1C). In a second step, a linear robust regression (lmrob) was applied on the same data sets (only in Participant 02, a reduced range of base-out prisms of 0, 2, 4 prism dioptre was used). These regressions were made for fixation disparity as a function of forced vergence curve, both subjective (sFD) and objective (oFD). An additional regression referred to sFD as a function of oFD. The linear robust regression has several advantages: (1) Outlying data and extreme values can occur due to random measurement error or due to extreme physiological conditions. Such data automatically receive a lower weight in a robust regression, without the need to consider each case separately. (2) The regression coefficients of the intercept and slope of fixation disparity curves are clinically known parameters to describe the individual status regarding the fixation disparity without a prism and the extent to which forced vergence changes the fixation disparity, respectively. (3) Individual confidence intervals of regression parameters allow for an estimation of the precision of the individual results.

On the group level, regression analyses across observers were made based on the individual regression parameters, i.e., the individual intercepts and slopes.

### Participants

The present study recruited participants with high visual acuity (without or with contact lenses) since spectacle lenses may prevent precise eye movement recording. The 12 young adult subjects aged 20–29 years (25.4 ± 2.6, mean ± SD). The far visual acuity–in decimal units–was generally 1.6 or better in each eye; only in one eye the acuity was 1.25. Two types of stereo tests were applied. The Polatest at 5 m includes small figural stereo targets: the average threshold was 50 ± 43 secarc (mean ± SD, range 30–180) for crossed disparity and 75 ± 78 secarc (30–120) for uncrossed disparity. The random-dot TNO test at 40 cm includes circles with missing sectors: the average threshold was 80 ± 29 secarc (60–120) for crossed disparity and 90 ± 54 secarc (60–240) for uncrossed disparity. The vergence ranges (amount of prism leading to double vision, break point) at 5 m was 1.5 ± 5.9 prism dioptre (2.5–8) for base-in prisms and 6.6 ± 14.3 prism dioptre (3.75–22) for base-out prisms. These optometric data generally refer to vision without glasses; only one myopic observer (- 2.5 and– 1.75 dioptre) wore contact lenses during all experiments.

This research was approved by the ethic committee of the Leibniz Research Centre of Working Environment and Human Factors (IfADo); the procedures were in accordance with ethical practice and participants signed a written consent.

## Results

### Individual fixation disparity curves

**Fig 4:**
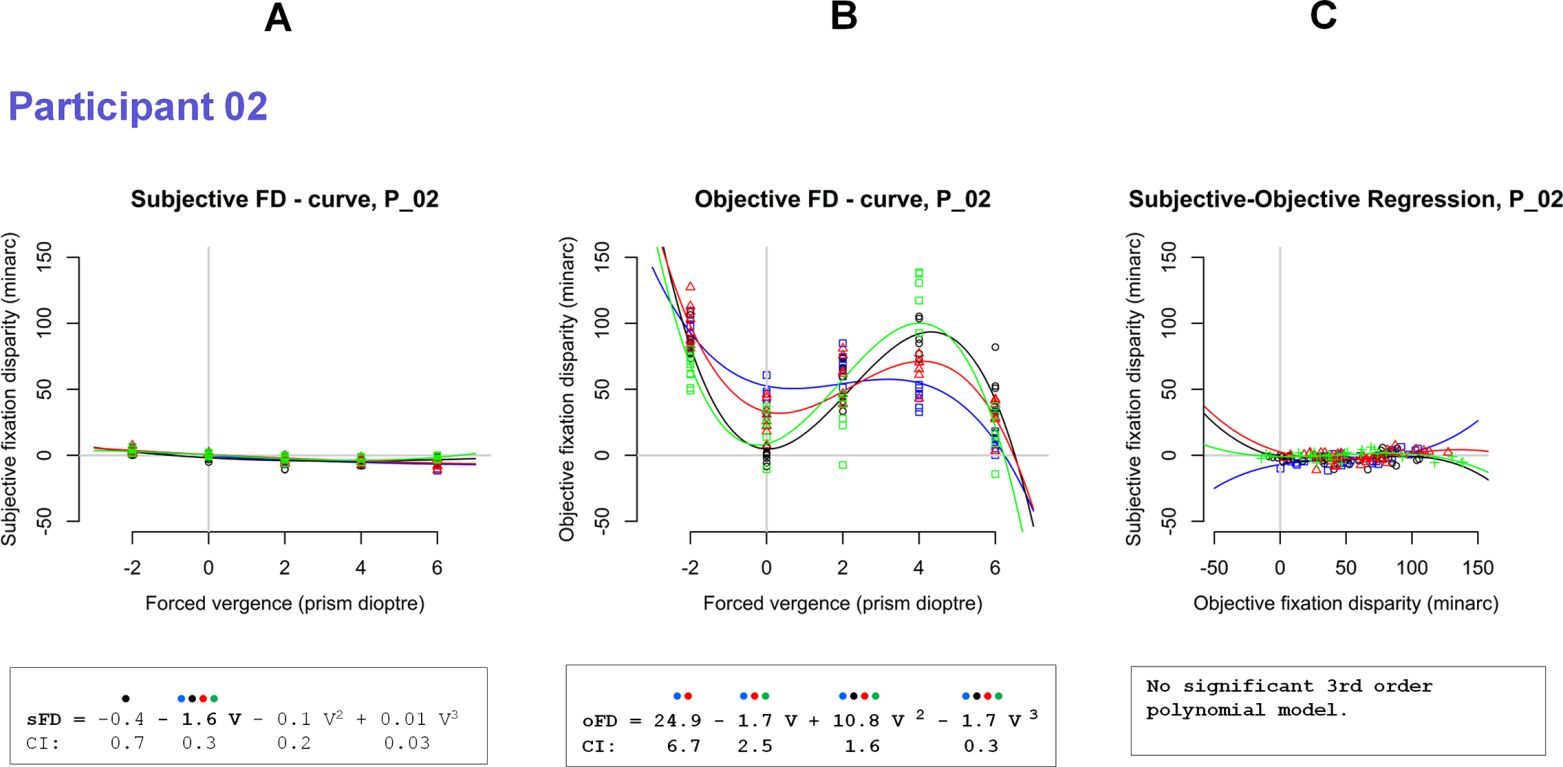
For Participant 2, the subjective (A) and objective (B) fixation disparity are plotted as a function of forced vergence, i.e. as a function of the prism load. The same scales are used for both types in order illustrate the different amount of these two measures. Below the regression equations of 3^rd^ order polynomials, the 95%-confidence intervals (CI) of the coefficients are given; if the confidence interval is smaller than the amount of the coefficient, the coefficient is significant (two-tailed) based on all four datasets of one individual; significant coefficients are printed bold. A coloured dot is drawn at each coefficient if this coefficient is significant in one of the four repeated measurements. Thus, four coloured dots mean that this coefficient is significant in each of the four plots. The data of (A) and (B) are replotted in (C) to show the subjective fixation disparity as a function of objective fixation disparity. The present data of Participant 02 show no significant regression model for sFD ~ oFD (but see other participants).

Fig 1C shows a typical monotonic fixation disparity curve with negative slope; the function is linear at smaller prisms and non-linear at larger prisms. The appropriate mathematical description is a 3^rd^ order polynomial regression that was applied in the first step to all individual curves. Fig 4 shows the result of Participant 02 who–surprisingly–revealed a large quadratic component for objective fixation disparity (Fig 4B) that was significant in all four repeated measurements (denoted by different colours): the oFD-curve had a local minimum without a prism and a local maximum at 4 prism dioptres in three of the four repeated measurements. In contrast, the sFD-curve for the same observer did not show such a quadratic component (Fig 4A): as expected, negative slopes were found consistently with significant linear coefficients in all four repetitions.

**Fig 5:**
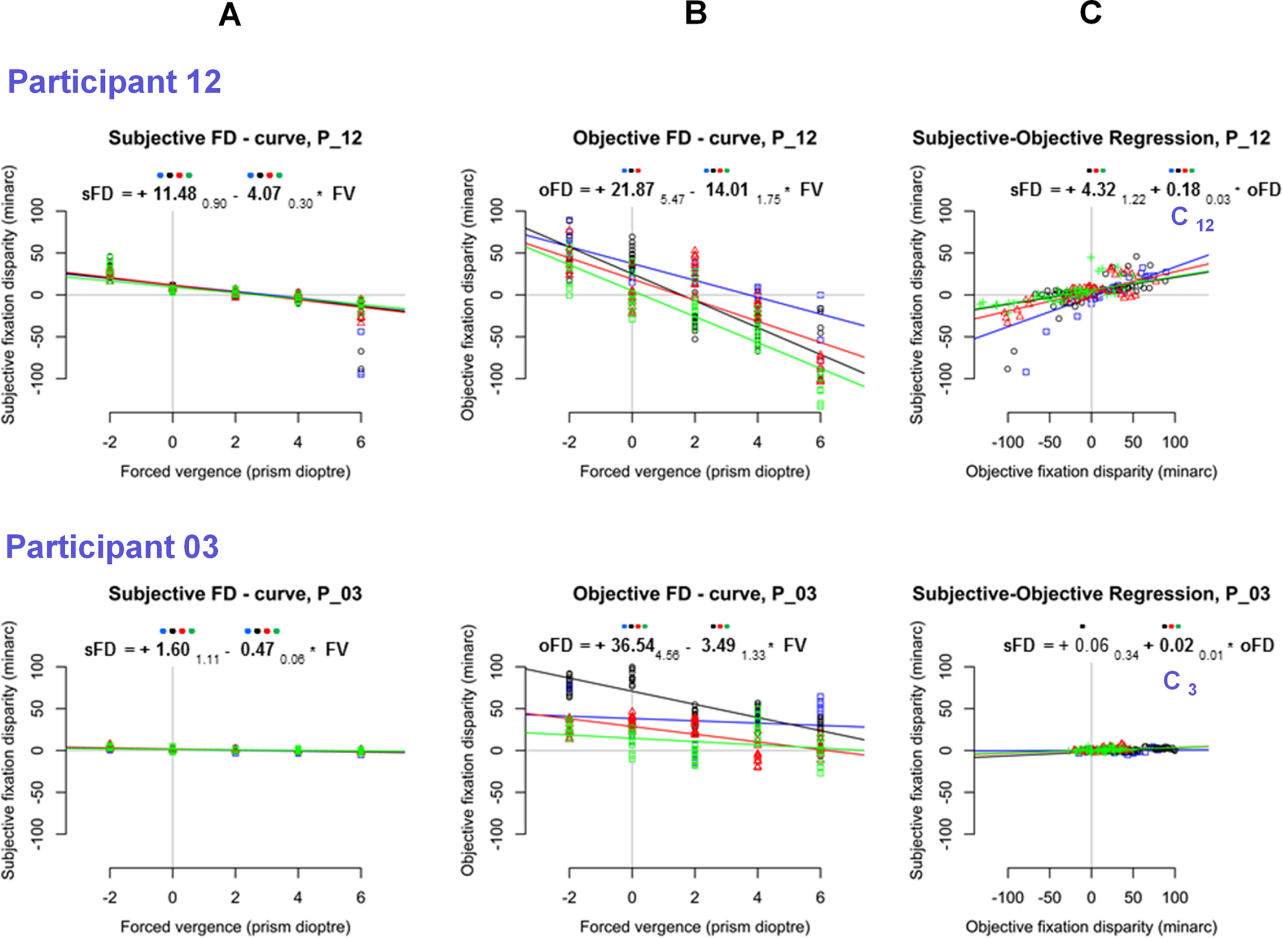
For Participants P12 and P03, the subjective (A) and objective (B) fixation disparity is plotted as a function of forced vergence, i.e., prism load. The same scales are used for both types of FD, in order illustrate the different amount of these two measures. The four colours represent the results of the four repeated measurements. Based on the combined data of all four repetitions, the equations describe the robust linear regressions lines with coefficients and 95%–confidence intervals (as subscripts); if the confidence interval (CI) is smaller than the amount of the coefficient, the coefficient is significant (two-tailed) based on all four datasets of one individual; significant coefficients are printed bold. A coloured dot is drawn at each coefficient if this coefficient is significant in one of the four repeated measurements. Thus, four coloured dots mean that this coefficient is significant in each of the four plots. The data of (A) and (B) are replotted in (C) for a regression of subjective fixation disparity as a function of objective fixation disparity, i.e., sFD_i j_ = k _i_ + C_i_ * oFD_i j_, when the single measurements j are plotted directly relative to each other for each individual i.

The analyses of the 3^rd^ order polynomials revealed that all other participants did not show reliable non-linear functions in the present range of prisms applied. Therefore, all other participants were analysed with a robust linear regression which provides estimations of the y–intercept (the value without applied prisms) and the slope (the change in the unit minutes of arc / prism dioptre). The latter are conventional parameters of fixation disparity curves. All individual plots are provided in S1 Fig. Fig 5 shows the data of two participants as examples of typical results. The data of Participant 12 in Fig 5A and 5B show rather large linear prism effects in both types of fixation disparity that were significant in all four repetitions. Without prisms, the fixation disparity was significantly positive. Generally, the variability and the amount of fixation disparity were larger in the objective than in the subjective case. In Fig 5A and 5B, the data of Participant 03 also show reliable and significant negative slopes, which were–however–smaller than for Participant 12.

S1 Fig shows that the data of 11 of all 12 participants allowed for a simple description of fixation disparity curves in terms of two parameters: the intercept (FD_0_, i.e., without prism) and linear slope (FD _slope_). In order to include also Participant 02 (Fig 4) into this scheme of a linear analysis, for this participant the analysis was limited to the linear part of the curve in the range from 0 to 4 prism dioptre, where the slope was positive (the linear analysis of Participant 02 is included in S1 Fig. This reduced linear analysis in Participant 02 will finally appear to fit into the linear pattern of results in the complete sample (See Discussion).

### Individual subjective versus objective regression

**Fig 6:**
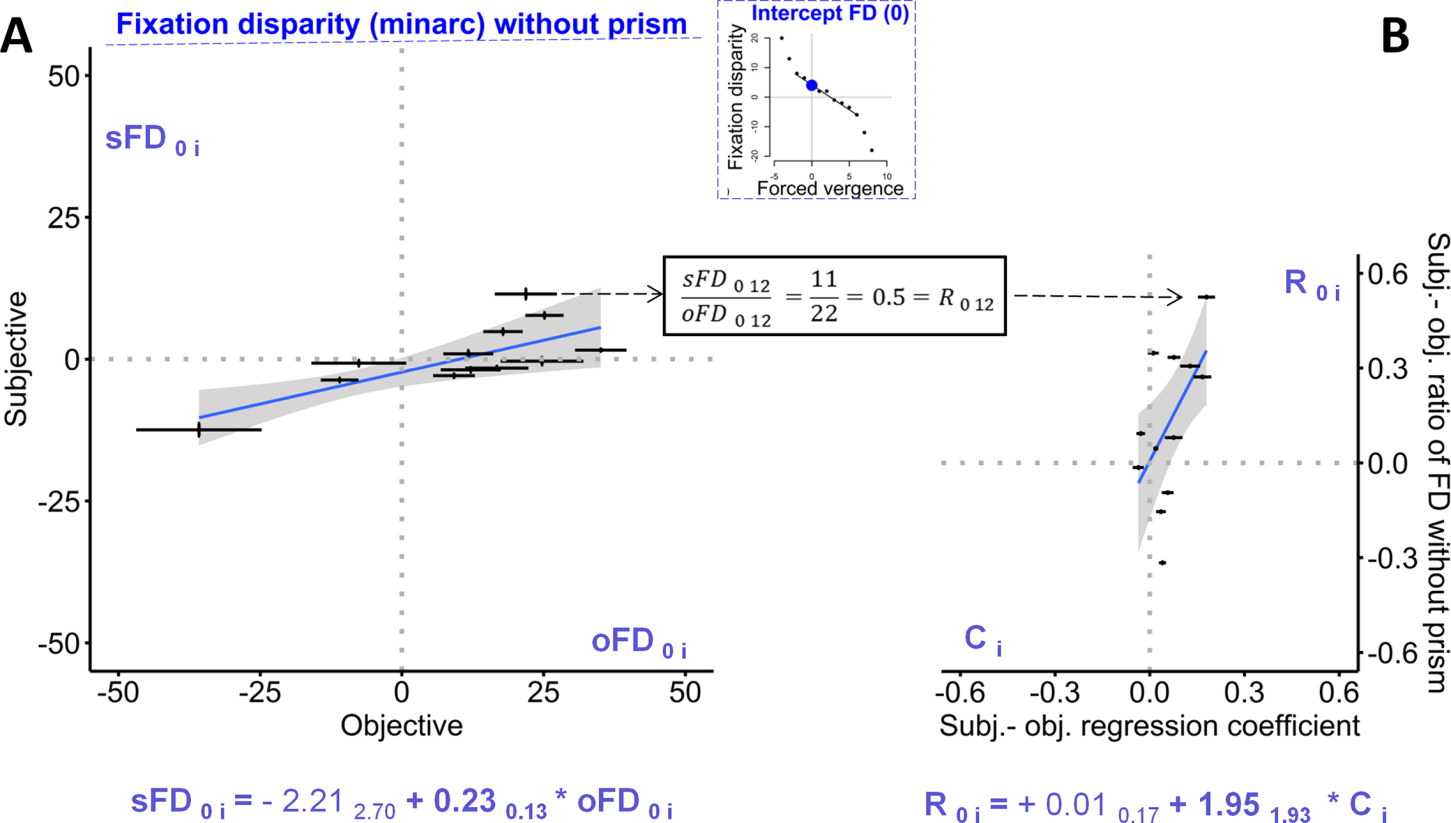
The left graph (A) shows the fixation disparity without forced vergence, i.e., without prism load, relating the subjective (sFD _o i_) to the objective measures (oFD _o i_): this is based on the 12 individual intercepts (and their confidence intervals shown as error bars) from the individual robust regressions (see Fig 5A and 5B and S1 Fig). For one case, the ratio R_0 12_ = sFD_0 12_ / oFD_0 12_ = 11 / 22 = 0.5 is illustrated, as one data point on the ordinate axis in Fig 6B which shows how the individual R_0 i_−ratios are predicted by the individual values of C _i_ (the individual coefficients resulting from regressions sFD_i j_ = C_i_ * oFD_i j_ across single data j recorded with varying forced vergence, see Fig 4C). Both graphs include the robust regression lines with the confidence ranges and the corresponding equations with coefficients and the confidence intervals as subscripts. For the R_0i –_ratios, a confidence interval is not directly available.

**Fig 7:**
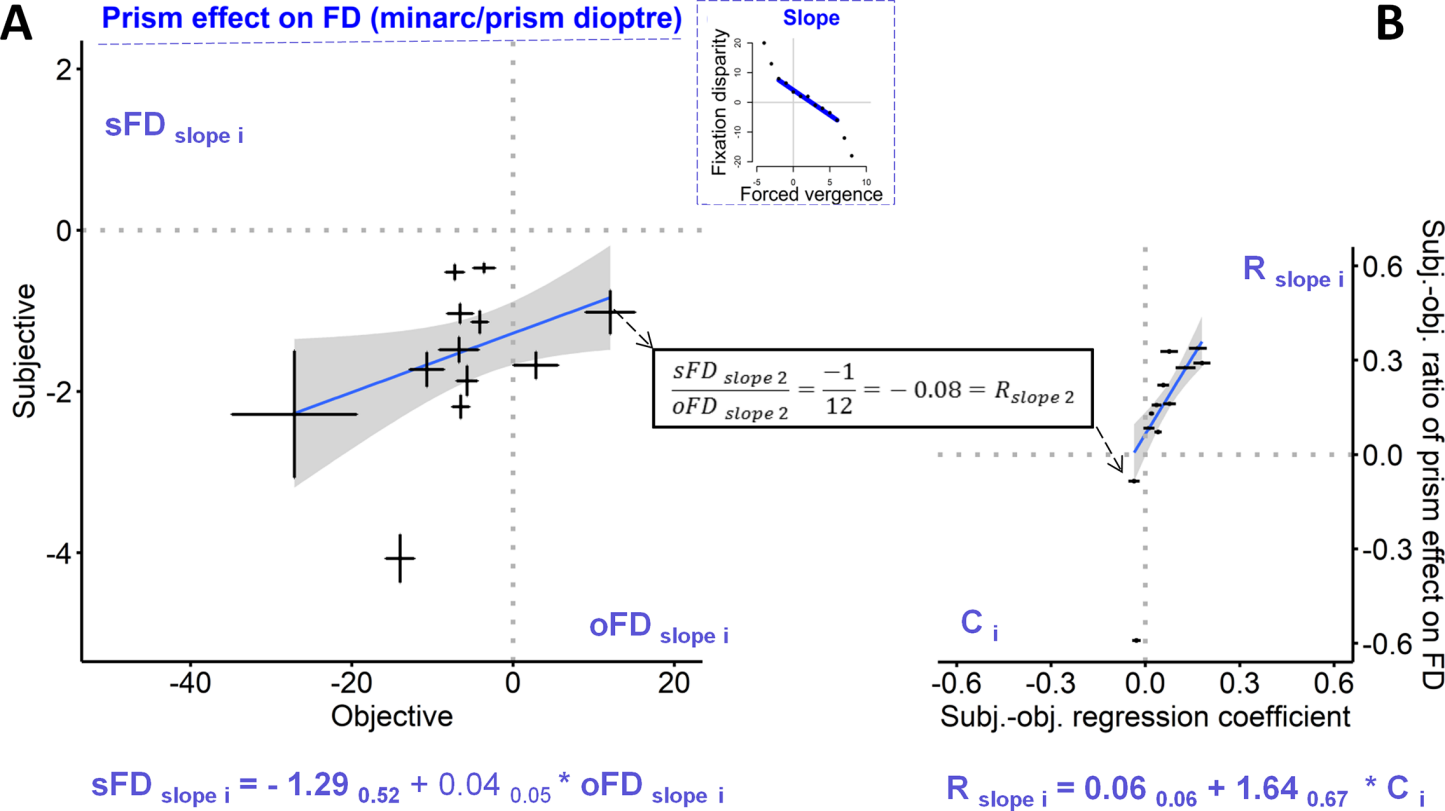
The left graph (A) shows the prism effect on fixation disparity, relating the subjective (sFD _slope i_) to the objective measures (oFD _slope i_): this is based on the 12 individual slopes (and their confidence intervals shown as error bars) from the individual robust regressions (see Fig 5A and 5B and S1 Fig). For one case, the ratio R_slope 2_ = sFD _slope 2_ / oFD _slope 2_ = – 1 / 12 = – 0.08 is illustrated, as one data point on the ordinate axis in Fig 7B, which shows how the individual values of R_slope i_ are predicted by the individual values of C _i_ (the individual coefficients resulting from regressions sFD_i j_ = C_i_ * oFD_ij_ across single data j recorded with varying forced vergence, see Fig 5C). Both graphs include the robust regression lines with the confidence ranges and the corresponding equations with coefficients and the confidence intervals as subscripts. For the R_slope i−_ratios, a confidence interval is not directly available.

The following analysis refers to the individual regression of subjective as a function of objective fixation disparity, i.e., sFD_i j_ = k _i_ + C_i_ * oFD_i j_, when for each individual i the single measurements j at varied forced vergence are related to each other. All individual regression plots are shown in S1 Fig; Fig 5C shows examples of Participant 12 and Participant 03 with coefficients C_12_ = 0.18 and C_3_ = 0.02, respectively, with the unit minarc/prism diopter. The individual coefficients C_i_ were significantly positive in 9 participants, as shown by the confidence intervals in Fig 6B and Fig 7B. Two exceptions were Participants 02 and Participant 09 with significantly negative coefficients of C_2_ = – 0.04 and C_6_ = – 0.03.

The constant terms k _i_ were expected to be zero, since one might have expected that sFD = 0 at oFD = 0. This was the case–however–in only two participants (Participant 03 in Fig 5C). All others had a significant constant term k _i_ meaning that a non-zero subjective fixation disparity occurred at oFD = 0, as in Participant 12 in Fig 5.

### Regressions on the group level

For further analyses, an individual mean and confidence interval was calculated for each participant in order to perform regression analyses on the group level. Fig 6 refers to the fixation disparity when no prism was applied, i.e., forced vergence was zero (FD_0_); these individual values of FD_0_ were calculated as the intercepts from the robust linear regressions as shown, as examples, in Fig 5A and 5B.

Fig 6A shows the relation between the subjective and the objective fixation disparity without prisms (sFD_0_ versus oFD_0_). The regression line has a slope of 0.23 minarc/prism dioptre that differed significantly from zero; the origin of the diagram is included in the confidence range, i.e., sFD_0_ = 0 at oFD_0_ = 0 on the group level. The individual confidence intervals are small enough to show that for most of the participants the individual confidence ranges do not include the regression line. This means that the ratio R_0 i_ = sFD_0 i_ / oFD_0 i_ (at prism = 0, for an observer i), is not constant in the sample, but varies between observers; this ratio is illustrated in Fig 6 for Participant 12 with R_0 12_ = 0.5. This suggests the hypothesis, that these individual R_0 i_−ratios at zero forced vergence may be related to the individual factors C _i_ that resulted from the regression sFD_j i_ = k _i_ + C _i_ * oFD _j i_ across all single data j at varying amounts of forced vergence, as shown in Fig 5C. In fact, Fig 6B shows this significant (p = 0.072 two-tailed, p = 0.036 one-tailed) regression, suggesting the relation R_0 i_ = 1.95 * C_i_ across all 12 participants; the corresponding intercept of 0.01 is negligible.

These analyses were also made for the slopes (FDC_slope_) of the fixation disparity curves, which describe the change in fixation disparity when forced vergence is introduced by prisms. Fig 7A shows the relation between the two types of slopes (sFD _slope_ and oFD _slope_), which did not reach significance (p = 0.14 two-tailed, p = 0.07 one-tailed). Nevertheless, the individual subjective-objective ratios of the two slopes were tested, i.e., R_slope i_ = sFD_slope i_ / oFD_slope i_; see the example of Participant 02 with a corresponding value of– 0.08. These R_slope i_−ratios were related to the C _i_−coefficients that resulted from the regression across individual single data j with varied amounts of prisms, i.e., sFD_i j_ = k_i_ + C _i_ * oFD_i j_. Fig 7B shows the significant relation of R_slope i_ = 0.064 _0.064_ + 1.64 _0.67_ * C _i_.

### The ratio R _i_ = sFD _i_ /oFD _i_ as a function of forced vergence

**Fig 8:**
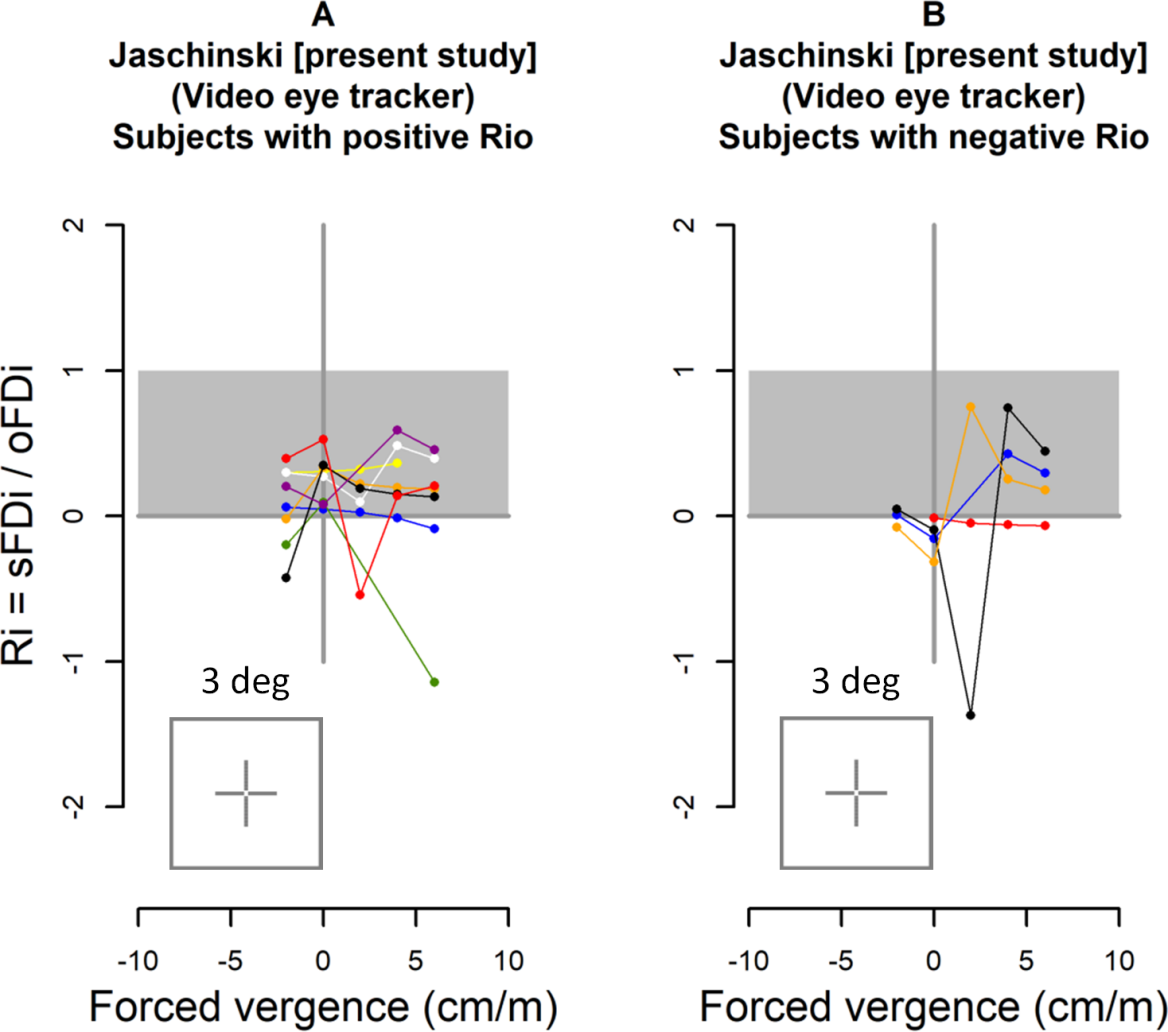
The ratio R_i_ = sFD _i_ /oFD_i_ is plotted as a function of forced vergence for each individual i. The gray area shows the expected range between 0 and 1 (see Introduction). Fig 8A includes the 8 participants with positive R_i0_ and Fig 8B includes the 4 participants with negative R_i0_, i.e., at zero forced vergence.

Fig 8 shows for each participant the ratio R_i_ = sFD _i_ /oFD_i_ at each amount of forced vergence. These data were calculated from the intercept and the slope of the individual forced vergence regression lines (see Fig 6 and Fig 7.). The complete sample was divided in two sub-samples. Fig 8A shows the eight participants with a positive ratio R_i_ at zero forced vergence; this was expected according to the physiological meaning of the ratio R_i_ (see Introduction). Fig 8B comprises the other 4 participants with a negative ratio R_i_ at zero forced vergence as it had appeared in Fig 6B. As Fig 8 shows, the data of the 4 observers in Fig 8B mostly fall within the distribution of the 8 observers in Fig 8A. The present small sample size does not allow confirming a statistically significant difference between these subsamples. Rather, in general the ratio R_i_ = sFD _i_/oFD_i_ seems to be more or less constant as a function of forced vergence within the expected positive R_i_ range in most subjects. Some participants had outlying data which can be explained by the definition of R_i_ = sFD _i_ /oFD_i_: when oFD approaches zero, R_i_−values approach infinity with a positive or negative sign, given that in these cases the experimental error will be larger than the amount of sFD or oFD. Because of this reason, 5 data points had been omitted for Fig 8, i.e. those cases in which oFD was virtually zero, i.e., when the oFD-values were within the range of ± one average confidence interval of oFD (which was 5.5 minarc). In these cases, the R_i_−values were 2.12, 2.69, 4.07, 2.20, -8.78, -1.98, which mostly were outside the plotted range in Fig 8. For the same reason, some outliers also occurred within the plotted range of 2 to– 2 (that agrees with the range plotted in Fig 2).

## Discussion

### Analyses of fixation disparity curves on the group level

The condition without prisms was analyzed as the y-intercept of the individual fixation disparity curves (FD_0_) which revealed that–on the group level–sFD_0_ was significantly dependent on oFD_0_ with a regression coefficient of 0.23 and an adjusted R^2^ = 0.50. Schroth et al. [5] also used the far-vision Cross test without central fusion stimulus and found similar regression parameters with a coefficient of 0.17 when no prisms were applied. In other studies with central fusion stimuli, this sFD vs. oFD regression coefficient tended to be smaller with 0.05–0.1 [5, 13]. Despite statistically significant regressions between sFD_0_ and oFD_0_, the explained variance is limited to about 50% in these studies. This is not the result of imprecise measurements as the present intra-individual confidence intervals suggest. Some individual data points deviate significantly from the group regression line which suggests that individuals differ in their relation between subjective and objective measures. This is the reason why a higher correlation is not found.

The effect of prisms is described by the individual slopes of fixation disparity curves (FD_slope_). Correlations between subjective and objective slopes on the group level have not been reported earlier and did not reach significance in the present study. See that in Fig 7 one can find 5 participants with similar objective slopes of about—6 minarc/prism dioptre, while the same participants differed significantly in their subjective slopes (as seen from the confidence intervals). The intra-individual confidence intervals were small enough so that a significant regression would have appeared if it had existed. All subjective slopes were negative (as expected), while two participants had objective positive slopes and both were significant as seen from their individual confidence intervals. These deviating cases are discussed below.

### Individual subjective versus objective fixation disparity

The core part of this study is the analysis of sFD as a function of oFD on the individual level. When many repeated simultaneous recordings of both measures were made at different amounts of prism load, the resulting individual sFD-oFD-regression coefficients C_i_ ranged between—0.04 and 0.17 in the sample, with an average of 0.08. As indicated by the individual confidence intervals, in 9 of the 12 participants these coefficients were statistically positive, but note that in 2 participants the coefficient was significantly negative. Thus, at least for most observers, the results provide evidence for the model sFD_ij_ = k_i_ + C_i_ * oFD_ij_, where i denotes the individual observer and j denotes different observations over time including different levels of prism load. The constant terms k_i_ are discussed below.

The regression coefficients C_i_ were significantly correlated with two other individual subjective-objective ratios. First, they were correlated with the R_slope i−_ratios which are the ratios of subjective versus objective slope of fixation disparity curves as a function of forced vergence (Fig 7B); this is expected since both the C_i−_coefficients and the R_slope i_−ratios include the effect of forced vergence. Second, more instructive is the correlation of the C_i_ –coefficients with the R_i 0 –_ratios since the C_i_−coefficients includes the effect of prisms, but the R_i 0_ –ratios reflect the state without prisms. Taken together, this suggests an individual subjective-objective relation, irrespective of whether prism effects are involved or not.

The approach of testing sFD_i_ = C_i_ * oFD_i_ on the individual level was recently proposed by Jaschinski [4] and tested based on the individual mean values of fixation disparity at viewing distances of 24, 30, and 40 cm. This did not reveal convincing patterns of results, presumably since the range of intra-individual variability was smaller than in the present study. However, Jaschinski [4] also re-analysed the published forced vergence data of the 9 subjects of Kertesz and Lee [27] and Fogt and Jones [12], who did not consider this individual approach: in 8 of these 9 subjects the two types of fixation disparity were well correlated (median r = 0.95, range 0.77 to 0.99); the sFD-oFD coefficients had a median of 0.58 (range 0.26 to 0.71). These coefficients in the studies of Kertesz and Lee [27] and Fogt and Jones [12] were larger than those in the present study.

### The effect of forced vergence on the R_i−_ratio

Remole et al. [24, 25] had introduced a measure of the ratio between subjective and objective fixation disparity, which he referred to as “projection change ration”, i.e. PCR = 1 –(sFD / oFD); see Introduction. Later research of Kertez and Lee [27] and Fogt and Jones [12] did not continue such kind of ratio analysis. The present study resumes this topic by defining (in the Introduction) a modified version of the ratio, i.e. R_i_ = sFD / oFD = 1 –PCR. Fig 2 provides a presentation of R_i_ which is based on the earlier published data of these authors [12, 24, 25, 27] and Fig 8 shows the corresponding results of the present study. For a comparison, one has to consider that the studies different in several respects, i.e., the spatial structure of central and peripheral fusion stimuli, the range of forced vergence, and the sample size. Moreover, different eye tracker recording techniques were used, as indicated.

From the definition, R_i_−values are expected within the range of 0 to 1. This is found in the large majority of data which generally confirms the hypothesis that sFD is between 0% and 100% of oFD, with individual differences. Compared to this expectation, some outliers occurred partly with negative data and partly with data much larger than 1. The general reason may be experimental error that is always included. A specific explanation lies in the definition of R_i_ = sFD/oFD, which means that R_i_ will increase (theoretically towards infinity) in the positive or negative direction, when oFD approaches zero. This effect appears to be strong in the first study of Remole [24] with the border enhancement effect.

Fig 2 and Fig 8 suggest that the results of the present study resemble those of the earlier eye tracker studies of Kertesz and Lee and Fogt and Jones: the R_i_−ratio of most individuals is rather constant across the divergent and divergent range (including zero prism load). The range of R_i_ ratios may be larger in the Fogt and Jones study, since these authors used the most peripheral fusion contour with a width of 6 deg, compared to 3.2 deg in the present study and a central fusion target in the Kertesz and Lee study. The results of Remole using the perceived border enhancement (with a central fusion stimulus) resembled those of the other studies when forced vergence was applied: small and positive R_i_−ratios were found. But near zero forced vergence, the perceived border enhancement resulted in R_i_−ratios that approached minus infinity in all participants. Such a deviating condition near zero forced vergence was not found in the three eye tracker studies. Thus, this may be due to specific viewing or test conditions of the perceived border enhancement technique.

### Visual directions of monocular and binocular objects

Given that the dichoptic nonius lines do not measure the inaccuracy of vergence (objective fixation disparity), the question arises, which may be the physiological mechanism involved in the perception of nonius lines that are presented monocularly to each eye.

The visual direction of a monocularly visible object has largely been investigated in studies of the rules how the visual directions of fused objects are processed by the visual system. For binocular objects, the general rules of cyclopean visual directions predict that the two fused binocular objects receive an averaged visual direction as if perceived from the cyclopean eye [40]. This rule cannot apply to monocular targets, since a counterpart in the fellow eye is missing. The monocular condition was studied in several approaches, including stationary forced vergence [12], dynamically changing absolute disparity [41], varying the relative disparity in random dot stereograms [42], or head movements [43]. Further, stimulus conditions were varied in terms of spatial frequency, contrast polarity, and vertical separation of monocular targets in order to relate the effects to mechanisms of relative position encoding.[44–46]. The latter studies provide reviews of this extended area of research. These considerably different approaches used partly different wording for the explanation of the findings. The general conclusion can be summarized as follows. Monocular targets are treated as binocular targets, if they are presented in close proximity to binocular targets [42]. This was referred to as a change in retinal correspondence [31], or capture of the visual direction of monocular targets by the visual direction of the close binocular targets [41]. This effect generally declines with an increasing separation between monocular and binocular targets.

These researches obviously refer to laboratory test conditions that were designed to test the rules of visual directions. One may wonder for which purpose the binocular visual system may have developed during evolution to reliably process monocular objects in natural vision. At first glance, monocular objects seem not to occur in natural vision where we always fuse the binocular targets that we intend to fixate. However, monocular contours occur whenever a part of a distant object is occluded for one eye by another closer object. Such conditions can occur very frequently, e.g., in a forest where trees and branches occlude each other. Then, binocular contours and monocular contours appear close together. The left and right eye images of the binocular contours are fused and receive the same visual direction by the sensory fusion mechanism. The monocular contours do not have a counterpart in the fellow eye so that conventional fusion cannot occur. The role of monocular contours in 3D-vision was the issue of more recent research [47–49] which has “shown that information from monocular regions is not simply thrown away by mechanisms dedicated to forming a seamless representation of the world. It is clear that monocular regions are important for forming surface representations and for depth perception. It is now possible to explain some phenomena that involve depth from monocular regions, using extensions of standard stereoscopic mechanisms. A parsimonious view would be that someday, all of these phenomena could be explained via elaborations of the binocular mechanisms that we know underlie standard disparity processing. But this has certainly not yet been demonstrated”(citation from the conclusion of Harris and Wilcox [47]). Concerning monocular targets in testing of subjective fixation disparity one could expect that the sensory mechanisms for natural 3D-vision will process—according to their inherent rules—the dichoptic nonius lines, although this stimulus does not include spatial depth. For this processing, the vergence error, i.e., the objective fixation disparity, seems to play a role in an idiosyncratic way, as the present study suggests.

### The question of zero fixation disparity

The hypothesis that sFD reflects a small percentage of oFD implies that sFD = 0 when oFD = 0. This, however, did only occur in 2 of the 12 participants where the intercept k_i_ of the individual regression sFD_i_ = k_i_ + C_i_ * oFD_i_ did not differ significantly from zero. In the 10 other participants, this intercept k_i_ was significantly negative in most cases. Therefore, the condition of zero fixation disparity needs particular attention. For an optimal comparison of sFD and oFD, the two definitions of zero fixation disparity should coincide in each individual. But this is questionable in two respects.

1. Concerning objective fixation disparity, oFD = 0 refers to the monocular calibration, when the fixation target is assumed to be projected onto the centre of the fovea in each eye. These retinal points correspond to the visual axes, that intersect in the stimulus plane in the case of oFD = 0. This calibration corresponds to the optometric definition of fixation disparity (Fig 1A) and is free from binocular influences. However, open questions remain. First, it is unknown whether the observer is fixating as intended during the calibration. One can only assume that the monocular calibration is a constant reference condition (as average across fixational eye movements) irrespective of forced vergence. Second, it is unclear whether this individual state of oFD = 0 is an optimal binocular status. Measurements of, e.g., stereo vision as a function of an experimental variation of oFD do not exist; but see the studies regarding the effect of a pedestal disparity of the stimulus on stereo threshold [50, 51] and vernier threshold [52]. Note further that a monocular calibration is an artificial viewing condition that does not occur in natural vision. Thus, the functional meaning of oFD = 0 is not self-evident and remains open at present. A binocular calibration, i.e., the fixation of binocular targets with both eyes during calibrations, is no solution since a fixation disparity during the calibration would be defined to zero; this would introduce a constant deviation in subsequent recordings [53, 54]. Thus, monocular calibrations are the best choice, but the implicit assumptions should be considered.
2. The subjective condition of sFD = 0 involves two questions. First, geometrically, sFD = 0 implies that physically aligned nonius targets appear in line when no binocular functions are involved, e.g., in monocular vision. This is the case if the two monocular targets are close to each other (as in the present study). But in other investigations the two nonius lines have a considerable vertical gap that includes a central fusion stimulus. Then, a small but reliable nonius offset of several minutes of arc can be perceived by many individuals, even if such two vertically separated nonius lines are perceived with one eye only. This phenomenon was referred to as “constant error” [55] or nonius bias [56]. Thus, it is uncertain whether sFD = 0 should be defined by physical nonius alignment or by the nonius bias [56]. Second, physiologically, the transfer of visual directions from binocular to monocular targets will be more accurate when both stimuli are closely adjacent, while larger separations may induce an uncertainty or bias. The present stimulus had a 1.6 deg separation between the central nonius task and the more peripheral fusion contour.

Thus, a valid and coinciding condition of sFD = 0 and oFD = 0 is difficult to verify experimentally in each individual. This may explain why 9 of the 12 present participants had significant non-zero amounts of sFD_i_ at oFD_i_ = 0 (based on the individual regressions); the range was -4.3 to 10.1 min arc. In some observers sFD and oFD can have even different signs: 3 participants had significant eso oFD and significant exo sFD. This uncertainty in the conditions of sFD = 0 and oFD = 0 is a principle limitation that needs to be taken into account.

### Deviating cases

The present sample of 12 observers was randomly chosen, with the only restriction to have good visual acuity in each eye without eye glasses. All data of all participants have been reported to show individual differences. Most participants had prism effects in the same direction as expected: sFD and oFD shifted monotonically to more exo conditions with increasing forced convergence due to base-out prisms. Participant 02, however, had a remarkably different response pattern as follows. When base-out prisms were increased from 0 to 4 prism dioptre, the eyes were required to exert a relative convergence up to 2.3 deg and showed a large objective eso shift with a concurrent subjective exo shift in fixation disparity. This contra-intuitive effect was reliable as confirmed by four repeated tests. Participant 06 also had slopes of different signs, but less reliable between repeated tests.

Interestingly, similar deviating cases were found by Jaschinski [4] when the convergence load was increased by shortening the viewing distance from 40 to 24 cm: generally, sFD shifted in the exo direction, while oFD shifted in the exo direction in some observers and–surprisingly—in the eso direction in others; thus, the average group effect was zero. In the present study, these two deviating cases can be interpreted regarding the group results. It is trivial that different signs of slopes give negative R_slope i_−values and negative C_i−_values (Fig 7B) since both refer to the prism effect. But it is informative that their R_0 i_−values (which are independent of the prism effect) fit into the distribution in the complete sample (Fig 6B). Thus, these two cases seem not to be complete outliers, but they may fit into the general response pattern in the sample. However, a full explanation cannot yet be provided.

More generally, individual differences in binocular coordination require particular research strategies including large sample sizes, repeated tests and a set of control variables. General rules based on mean values might not be applicable to each individual.

### Clinical interpretations and caveats

Clinicians, who measure sFD but not oFD, might be interested in potential conclusions and interpretation concerning the oculomotor oFD.

If sFD is large, oFD tends to be large as well. Thus, the sensory fusion mechanism did not achieve a full transfer of visual direction from binocular to monocular targets when a large oculomotor vergence error was present. When sFD = 0 occurs without prisms (or has been reached by means of prisms), this could mean two conditions: (1) oFD ≠ 0 and sensory fusion was fully effective to transfer the visual direction from binocular targets to monocular targets, or (2) oFD = 0 and sensory fusion is not challenged. Thus, a clinically tested sFD = 0 does not allow for clear diagnosis with respect to the motor and sensory conditions. More concretely, the stimulus in the present study, the Cross-test, was supposed to test primarily the oculomotor condition of fixation disparity [33–35, 37]; this seems questionable since a value of sFD = 0 does not allow to conclude that oFD = 0 [57, 58]

Concerning the individual slope values, the sFD-slope does not allow for prediction of the oFD-slope. Note that subjects with a very similar average oFD-slope of– 8 minarc/prism dioptre have significantly different slope values in range from– 0.5 to– 2 minarc /prism dioptre. Given that clinical studies have shown that the sFD-slope tends to be associated with asthenopia[17], one may consider whether the sensory mechanism of sFD may be associated with asthenopia. Note however, that studies of asthenopia versus oFD have not yet been made.

### Benefits, limitations, future perspectives

In terms of methodology, the present study showed for the first time, that video eye tracker procedures are useful for investigating the objective fixation disparity curves on the individual level. Such a methodology is a prerequisite for further clinically related studies of binocular coordination where individual diagnoses are required.

The present findings are only a first step, since the conclusions refer to the particular viewing condition, i.e., a fusion stimulus without a central fusion target in far vision. Moreover, the prisms were applied only for a few seconds, which is typical for measuring fixation disparity curves. It can be expected that the amount of fixation disparity and the effect of prism effects may be smaller with central fusion stimuli and when the prisms are applied for longer periods when prism adaptation may occur. The present Cross test is clinically used in the MCH-procedure [33–35, 37]; other important clinical tests refer to near vision at 40 cm and include a central fusion stimulus [19, 20]. These conditions need to be investigated with a similar approach.

Finally, the terms “objective and subjective fixation disparity” are used in research since many decades. Still, these terms can be an obstacle for the understanding of the physiological mechanisms. These terms may suggest that they refer to the same visual function which are only measured with different method and–further–these terms may suggest that the objective measure is a more valid measure and subjective measure being less relevant or even useless of clinical purposes. Such interpretations are not justified. Rather, researchers and clinicians should consider the different properties. Consequently, one may even consider using different terms, as “vergence error” and “(dichoptic) nonius offset”.

## Supporting information

S1 Fig The individual linear fixation disparity curves of all participants. (PDF)

S1 File Data for individual analyses. (XLSX)

S2 File Data for group analyses. (XLSX)
