# Supplementary material for: Individual objective versus subjective fixation disparity as a function of forced vergence
Source: PLoS One. 2018 Jul 6;13(7):e0199958. doi: 10.1371/journal.pone.0199958 (PMC6035046; doi:10.1371/journal.pone.0199958)
Supplement: S1 Fig — (PDF) [file pone.0199958.s001.pdf]

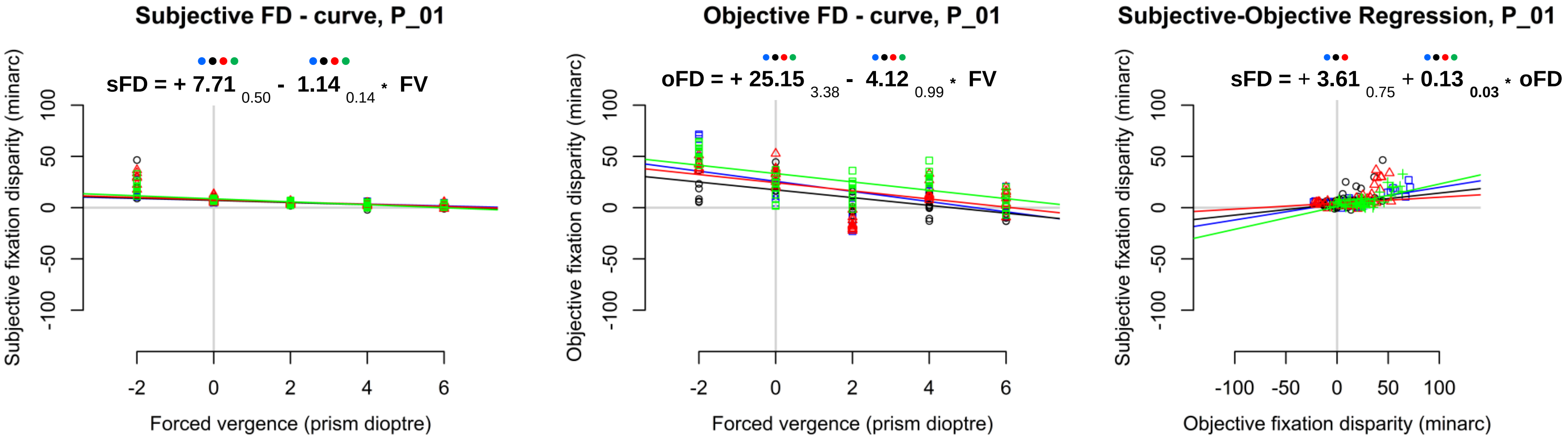
Subjective FD - curve, P_01
sFD = + 7.71 0.50 - 1.14 0.14 * FV
Subjective fixation disparity (minarc)
Forced vergence (prism dioptre)
Objective FD - curve, P_01
oFD = + 25.15 3.38 - 4.12 0.99 * FV
Objective fixation disparity (minarc)
Forced vergence (prism dioptre)
Subjective-Objective Regression, P_01
sFD = + 3.61 0.75 + 0.13 0.03 * oFD
Subjective fixation disparity (minarc)
Objective fixation disparity (minarc)

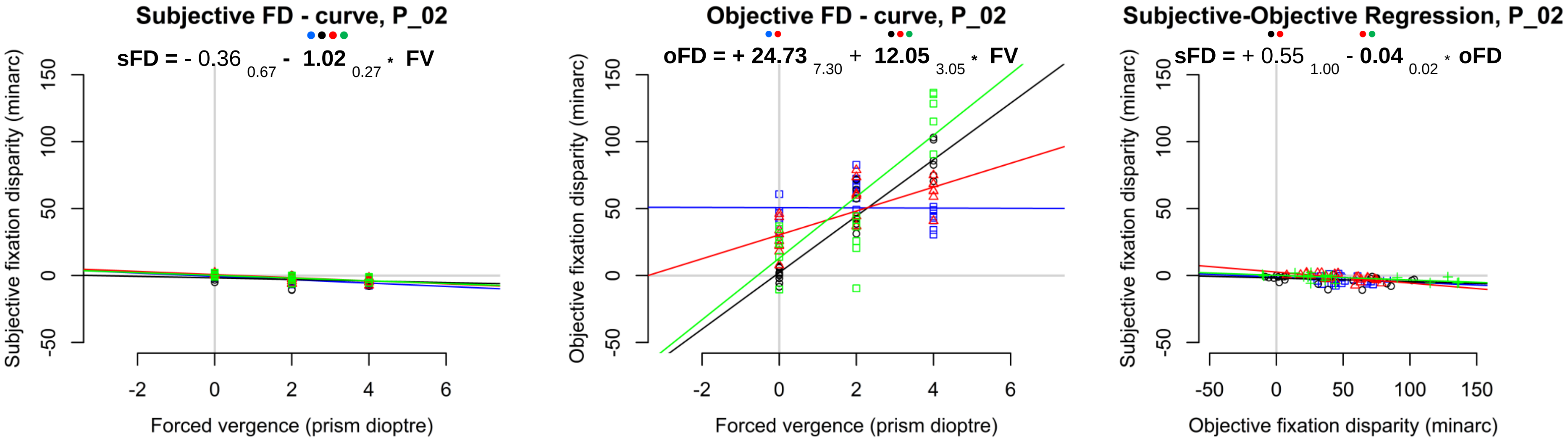

Subjective FD - curve, P_02
sFD = - 0.36 0.67 - 1.02 0.27 * FV
Subjective fixation disparity (minarc)
Forced vergence (prism dioptre)
Objective FD - curve, P_02
oFD = + 24.73 7.30 + 12.05 3.05 * FV
Objective fixation disparity (minarc)
Forced vergence (prism dioptre)
Subjective-Objective Regression, P_02
sFD = + 0.55 1.00 - 0.04 0.02 * oFD
Subjective fixation disparity (minarc)
Objective fixation disparity (minarc)

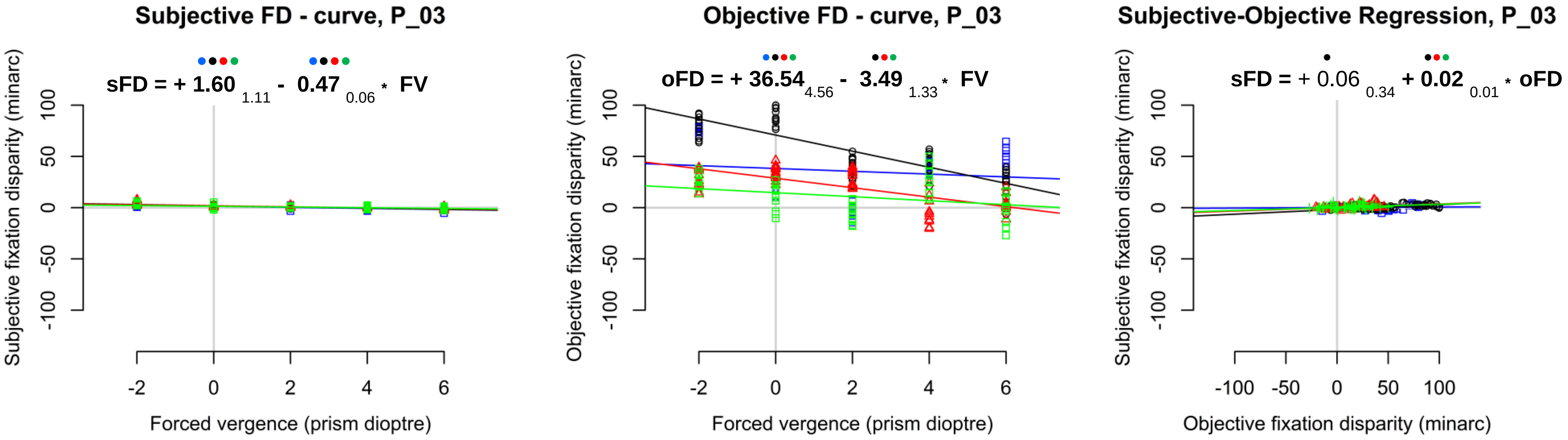

Subjective FD - curve, P_03
sFD = + 1.60 1.11 - 0.47 0.06 * FV
Subjective fixation disparity (minarc)
Forced vergence (prism dioptre)
Objective FD - curve, P_03
oFD = + 36.54 4.56 - 3.49 1.33 * FV
Objective fixation disparity (minarc)
Forced vergence (prism dioptre)
Subjective-Objective Regression, P_03
sFD = + 0.06 0.34 + 0.02 0.01 * oFD
Subjective fixation disparity (minarc)
Objective fixation disparity (minarc)

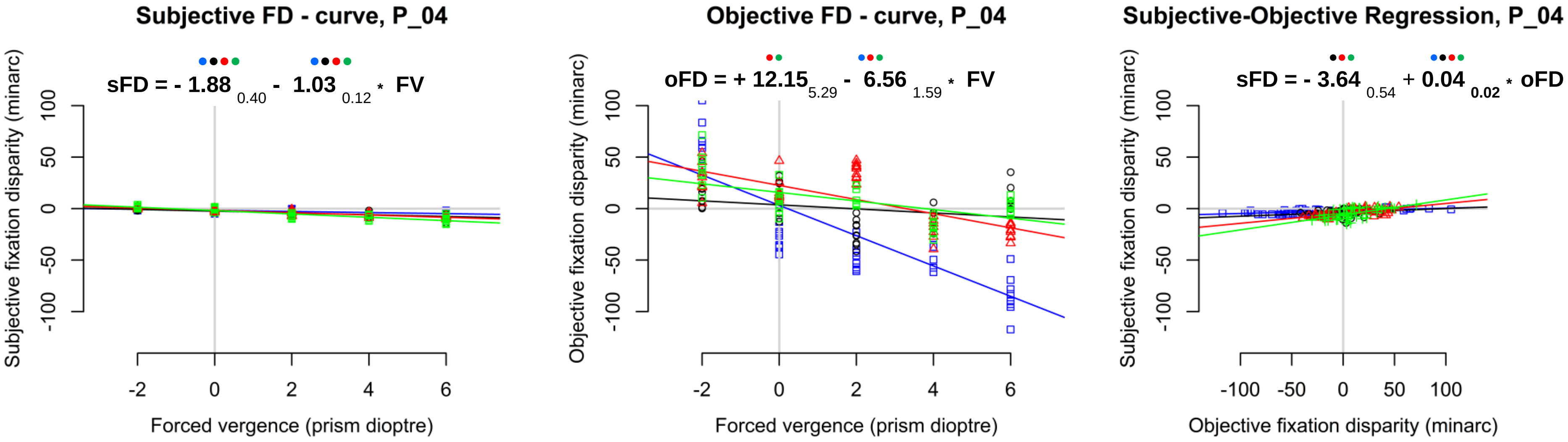
Subjective FD - curve, P_04
sFD = - 1.88 0.40 - 1.03 0.12 * FV
Subjective fixation disparity (minarc)
Forced vergence (prism dioptre)
Objective FD - curve, P_04
oFD = + 12.15 5.29 - 6.56 1.59 * FV
Objective fixation disparity (minarc)
Forced vergence (prism dioptre)
Subjective-Objective Regression, P_04
sFD = - 3.64 0.54 + 0.04 0.02 * oFD
Subjective fixation disparity (minarc)
Objective fixation disparity (minarc)

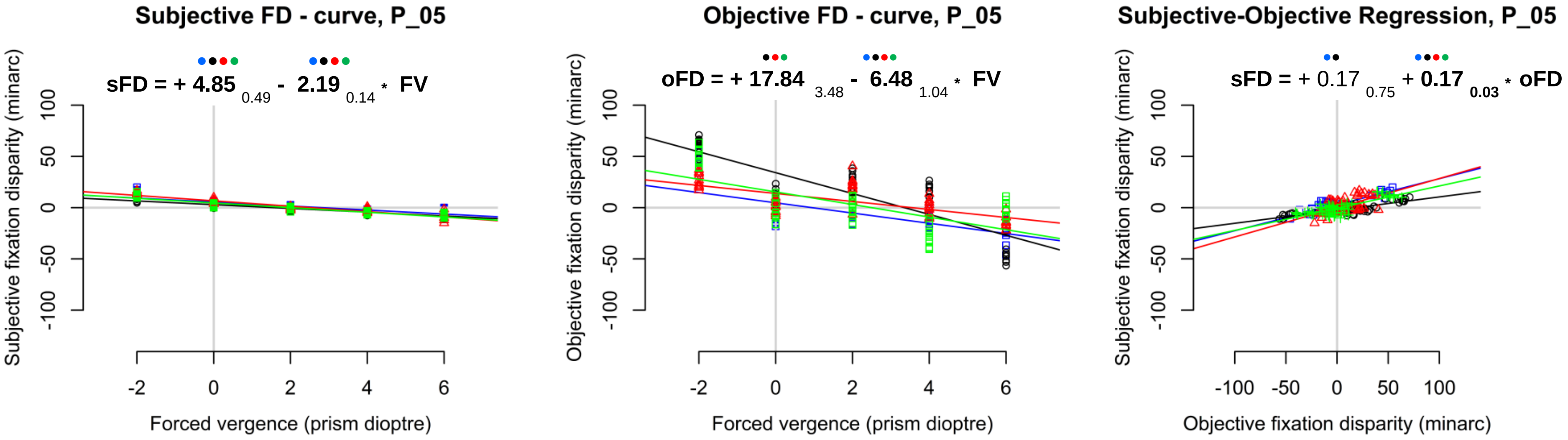
Subjective FD - curve, P_05
sFD = + 4.85 0.49 - 2.19 0.14 * FV
Subjective fixation disparity (minarc)
Forced vergence (prism dioptre)
Objective FD - curve, P_05
oFD = + 17.84 3.48 - 6.48 1.04 * FV
Objective fixation disparity (minarc)
Forced vergence (prism dioptre)
Subjective-Objective Regression, P_05
sFD = + 0.17 0.75 + 0.17 0.03 * oFD
Subjective fixation disparity (minarc)
Objective fixation disparity (minarc)

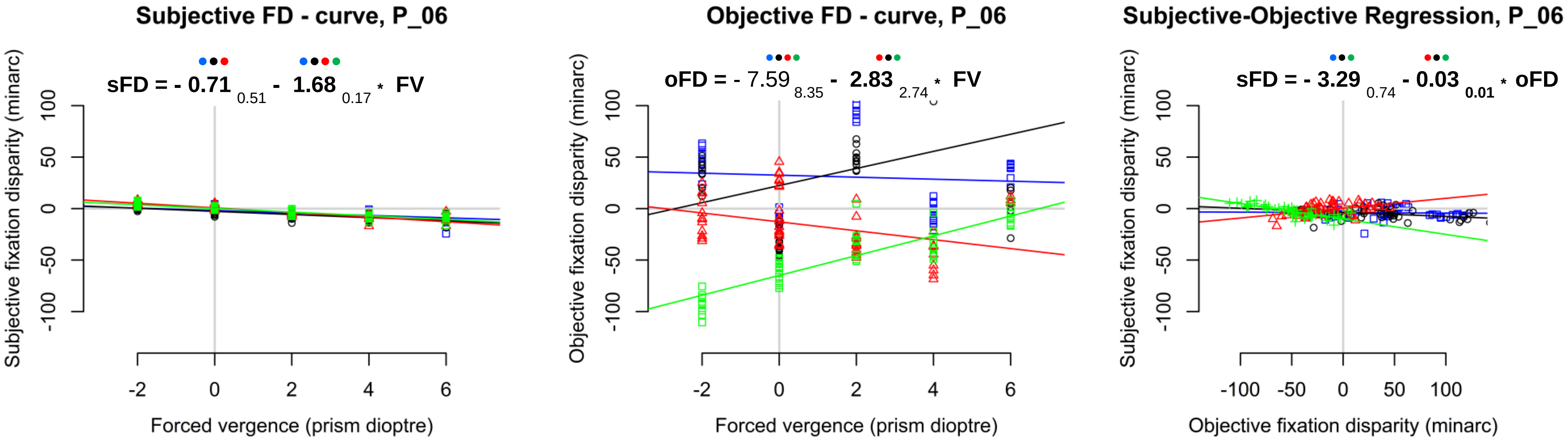

Subjective FD - curve, P_06
sFD = - 0.71 0.51 - 1.68 0.17 * FV
Subjective fixation disparity (minarc)
100
50
0
-50
-100
-2
0
2
4
6
Forced vergence (prism dioptre)
Objective FD - curve, P_06
oFD = - 7.59 8.35 - 2.83 2.74 * FV
Objective fixation disparity (minarc)
Forced vergence (prism dioptre)
Subjective-Objective Regression, P_06
sFD = - 3.29 0.74 - 0.03 0.01 * oFD
Subjective fixation disparity (minarc)
Objective fixation disparity (minarc)

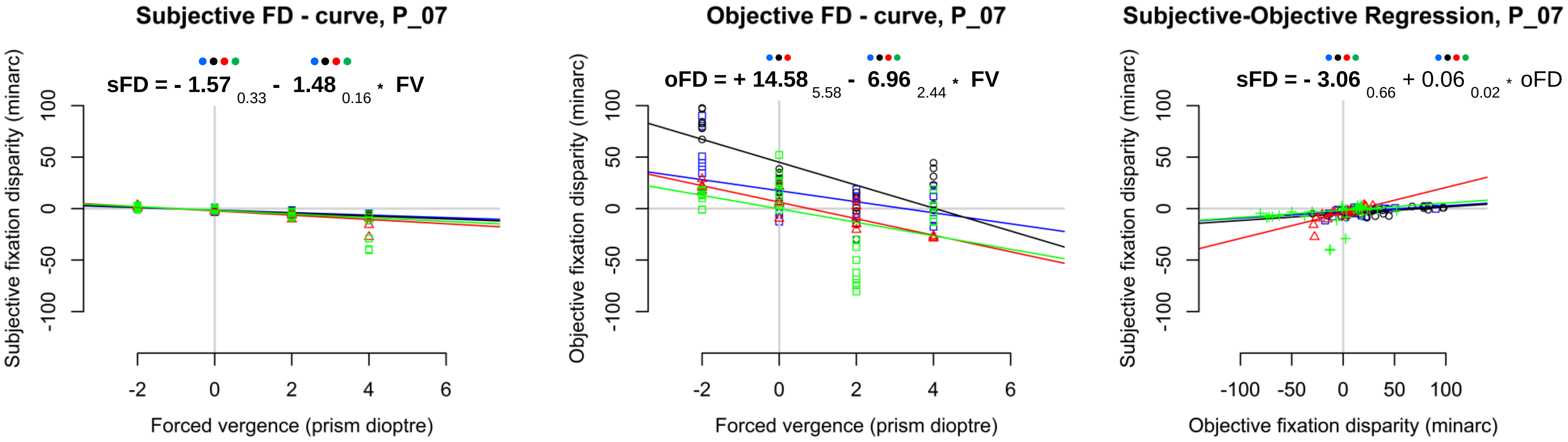

Subjective FD - curve, P_07
sFD = - 1.57 0.33 - 1.48 0.16 * FV
Subjective fixation disparity (minarc)
100
50
0
-50
-100
-2
0
2
4
6
Forced vergence (prism dioptre)
Objective FD - curve, P_07
oFD = + 14.58 5.58 - 6.96 2.44 * FV
Objective fixation disparity (minarc)
Subjective-Objective Regression, P_07
sFD = - 3.06 0.66 + 0.06 0.02 * oFD
-100
-50
50
100
Objective fixation disparity (minarc)

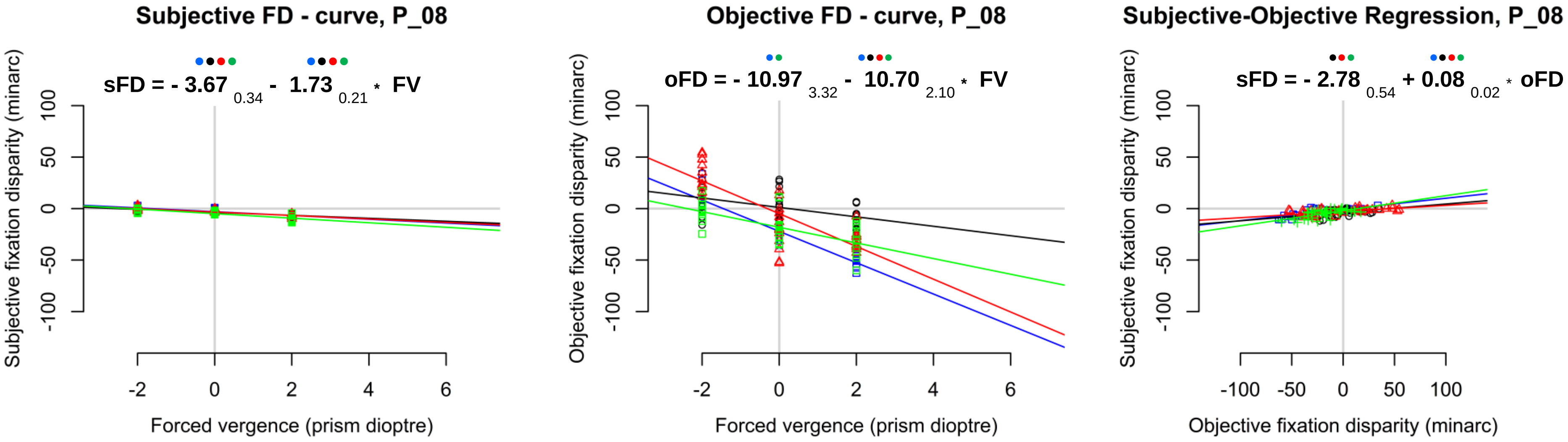

Subjective FD - curve, P_08
sFD = - 3.67 0.34 - 1.73 0.21 * FV
Subjective fixation disparity (minarc)
Forced vergence (prism dioptre)
Objective FD - curve, P_08
oFD = - 10.97 3.32 - 10.70 2.10 * FV
Objective fixation disparity (minarc)
Forced vergence (prism dioptre)
Subjective-Objective Regression, P_08
sFD = - 2.78 0.54 + 0.08 0.02 * oFD
Subjective fixation disparity (minarc)
Objective fixation disparity (minarc)

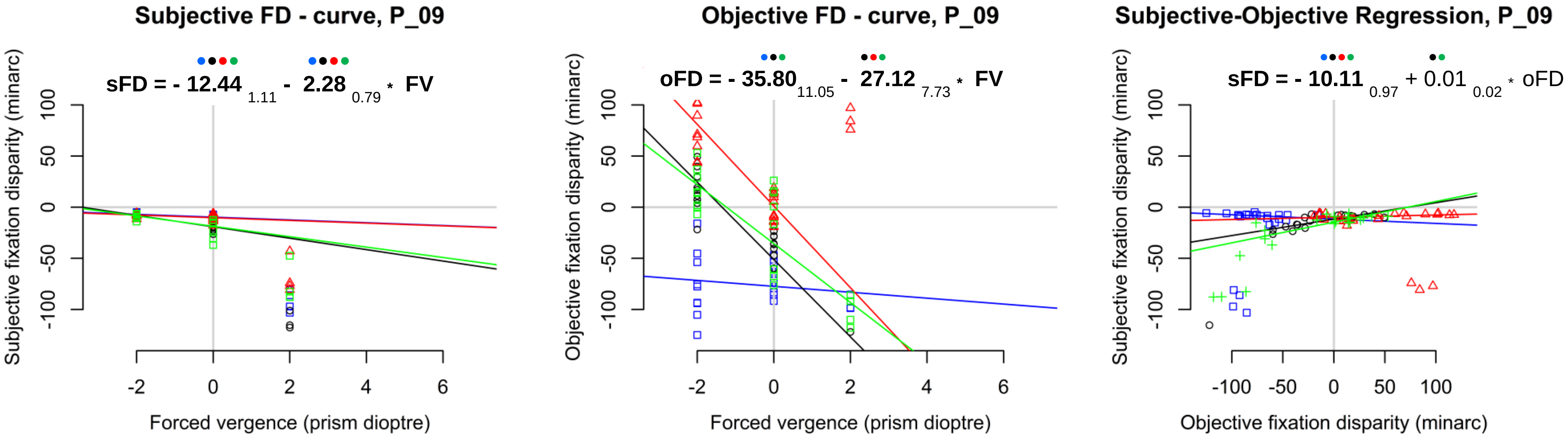

Subjective FD - curve, P_09
sFD = - 12.44 1.11 - 2.28 0.79 * FV
Subjective fixation disparity (minarc)
Forced vergence (prism dioptre)
Objective FD - curve, P_09
oFD = - 35.80 11.05 - 27.12 7.73 * FV
Objective fixation disparity (minarc)
Forced vergence (prism dioptre)
Subjective-Objective Regression, P_09
sFD = - 10.11 0.97 + 0.01 0.02 * oFD
Subjective fixation disparity (minarc)
Objective fixation disparity (minarc)

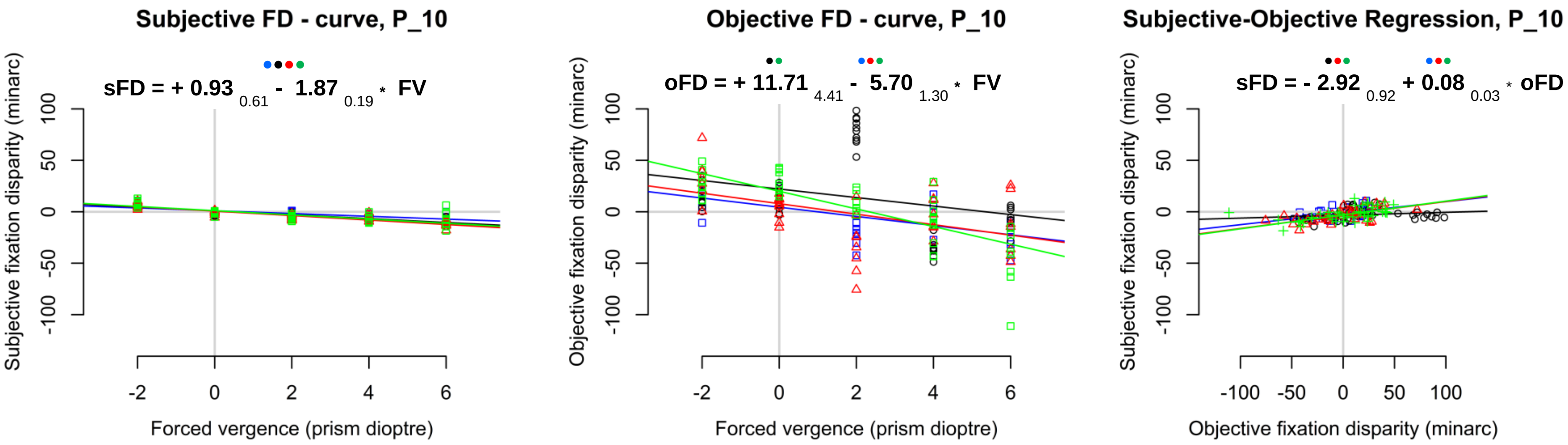

Subjective FD - curve, P_10
sFD = + 0.93 0.61 - 1.87 0.19 * FV
Subjective fixation disparity (minarc)
Forced vergence (prism dioptre)
Objective FD - curve, P_10
oFD = + 11.71 4.41 - 5.70 1.30 * FV
Objective fixation disparity (minarc)
Forced vergence (prism dioptre)
Subjective-Objective Regression, P_10
sFD = - 2.92 0.92 + 0.08 0.03 * oFD
Subjective fixation disparity (minarc)
Objective fixation disparity (minarc)

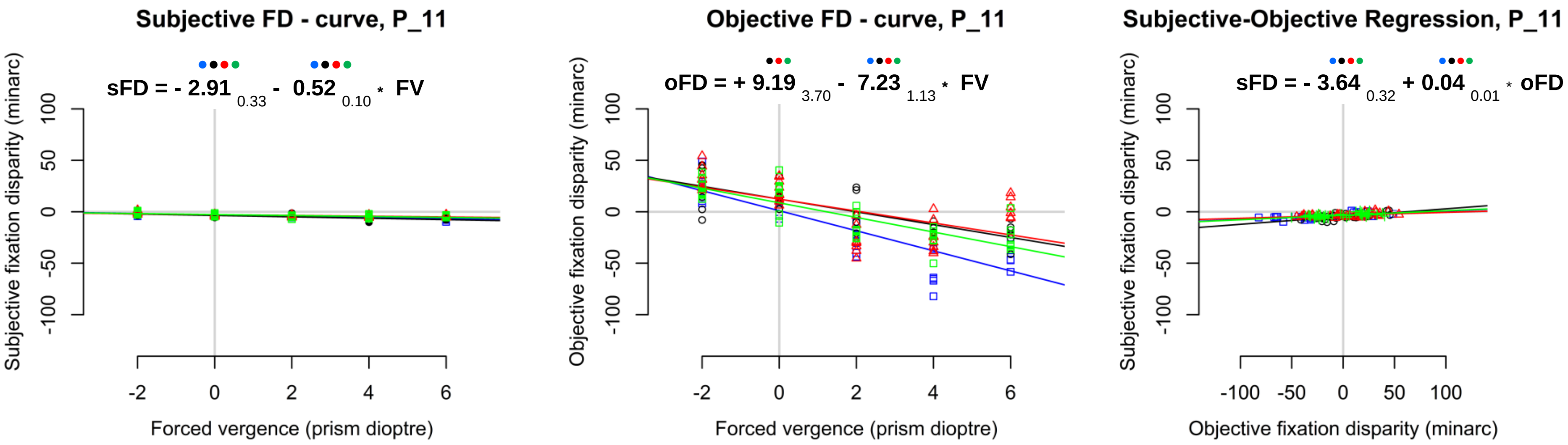
Subjective FD - curve, P_11
sFD = - 2.91 0.33 - 0.52 0.10 * FV
Subjective fixation disparity (minarc)
100
50
0
-50
-100
-2
0
2
4
6
Forced vergence (prism dioptre)
Objective FD - curve, P_11
oFD = + 9.19 3.70 - 7.23 1.13 * FV
Objective fixation disparity (minarc)
100
50
0
-50
-100
-2
0
2
4
6
Forced vergence (prism dioptre)
Subjective-Objective Regression, P_11
sFD = - 3.64 0.32 + 0.04 0.01 * oFD
Subjective fixation disparity (minarc)
100
50
0
-50
-100
-100
-50
0
50
100
Objective fixation disparity (minarc)

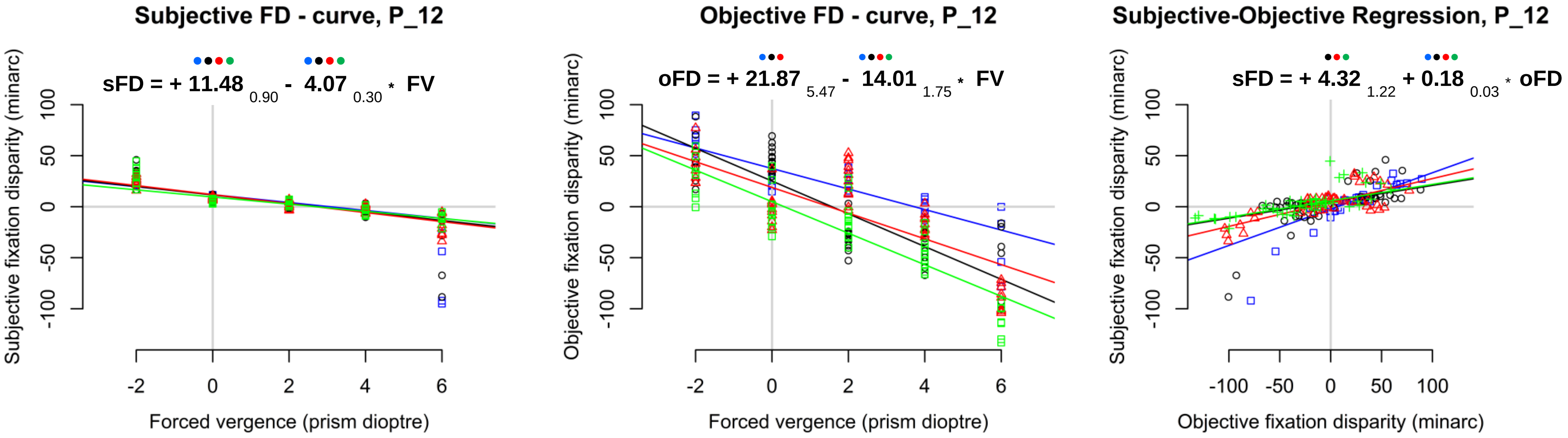
Subjective FD - curve, P_12
sFD = + 11.48 0.90 - 4.07 0.30 * FV
Subjective fixation disparity (minarc)
Forced vergence (prism dioptre)
Objective FD - curve, P_12
oFD = + 21.87 5.47 - 14.01 1.75 * FV
Objective fixation disparity (minarc)
Forced vergence (prism dioptre)
Subjective-Objective Regression, P_12
sFD = + 4.32 1.22 + 0.18 0.03 * oFD
Subjective fixation disparity (minarc)
Objective fixation disparity (minarc)
